# Supplementary material for: Implementation of the NHS England Lung Cancer Screening Programme over 5 years
Source: Nat Med. 2026 Mar 23;32(5):1817–26. doi: 10.1038/s41591-026-04292-y (PMC13190264; doi:10.1038/s41591-026-04292-y)
Supplement: Supplementary file 1 — Standard Protocol and Quality Assurance Standard. [file 41591_2026_4292_MOESM1_ESM.pdf]

nature medicine

Supplementary information https://doi.org/10.1038/s41591-026-04292-y

Implementation of the NHS England Lung Cancer Screening Programme over 5 years

In the format provided by the authors and unedited

Classification: Official

Publication reference: PR1646

NHS England

Targeted screening for lung cancer with low radiation dose computed tomography

Standard protocol prepared for the Lung Cancer Screening Programme

Version 3, 3 February 2025

Prepared with guidance from the Lung Cancer Screening Programme Clinical Expert Advisory Group

Contents

1. Background and introduction 2

2. Clinical governance..6

3. Assessment process 11

4. Low dose computed tomography acquisition and reading 24

Repeat low dose computed tomography ....**Error! Bookmark not defined.**

5. Surveillance of indeterminate nodules (pathway B)..30

Management of findings ....**Error! Bookmark not defined.**

6. Pathway A: Findings requiring further investigation..38

7. Non-attendance, moving out of area and exiting the programme.....................41

8. Communication of results 44

9. Low dose computed tomography data management.......................................46

10. Nonadherence with Standard Protocol and governance documents................47

11. Evolution of the standard protocol for the LCSP...48

References 49

Appendix A 50

Acknowledgements...51

1. Background and introduction

1.1 Targeted screening for lung cancer standard protocol

1.1.1 The purpose of this standard protocol is to ensure that there is a high quality, consistent and equitable approach to the provision and monitoring of targeted screening for lung cancer across England. This protocol covers the pathway from cohorting and invitation to suspected cancer.

1.1.2 This document is designed to outline the minimum and recommended service expected by NHS England to ensure that a high and consistent standard of service is provided.

1.1.3 This document sits alongside the required Quality Assurance Standards and UKNSC Lung Screening Standards by which individual Lung Cancer Screening sites are assessed.

1.1.4 The Lung Cancer Screening Programme (LCSP) is the national lung cancer screening programme for England. It falls within the UK National Screening Committee definitions of targeted and stratified national screening (UK NSC: evidence review process - GOV.UK) The Lung Cancer Screening Programme (now Lung Cancer Screening) was noted to be a feasible and effective starting point for implementation in England (Lung cancer - UK National Screening Committee .All sites participating in the Lung Cancer Screening Programme (LCSP) must adhere to this standard protocol for targeted lung cancer screening.

1.2 Definitions

1.2.1 Although targeted screening for lung cancer and population-based screening follow the same basic principles, they differ because targeted screening uses additional risk factors beyond age and sex to define the eligible population with a higher risk of a specific condition. Lung cancer screening uses smoking history as an additional risk factor. Once invited, participant risk is assessed using multivariable mathematical models.

1.2.2 Stratified screening is a national programme where the frequency or modality of screening may be varied according to risk. In the LCSP,

screening interval is altered according to the presence of pulmonary nodules, which alters the risk of lung cancer developing.

1.2.3 The Lung Cancer Screening Programme selects participants from eligible populations at risk of lung cancer due to smoking history and offers LDCT to higher risk participants.

1.2.4 Until full national rollout is complete, LCSP will be overseen by the NHSE cancer programme including the LCSP Expert Advisory Group.

1.2.5 Programmes may include other health interventions as long as they do not conflict with the Standard Protocol and as long as funding for lung cancer screening is not used to provide them . These may focus on lung cancer prevention, and/or in the diagnosis/management of other conditions. It is important that this does not hinder lung cancer screening and is either evidence based or part of approved research.

1.3 Aims

1.3.1 The primary aim is to reduce mortality from lung cancer. This must be achieved with minimum physical and psychological harm. To do this, the programmes should be delivered to meet or exceed nationally set standards and pathways that:

- define who should be invited (the cohort)
- have robust (electronic) mechanisms to invite the cohort and recall for those who require surveillance or a routine screen after an interval
- include measures to improve uptake and reduce inequalities (while honouring the principle of informed choice), e.g. ensuring tests are carried out and results are communicated in a format that is most appropriate for the individual participant
- provide appropriate information for participants to allow them to make an informed choice about participating, including recognition of any risks associated with the test itself and possible outcomes, such as referral for invasive procedures and associated risks
- describe the tests to be carried out
- define the results of the tests including positive (abnormality), negative and indeterminate

- describe (or points at) the follow up diagnostic and treatment pathways according to best practice (e.g. NICE, British Thoracic Society [BTS] guidelines) for all categories of tests and their results (including negative)
- are delivered and supported by clinical and non-clinical staff who are suitably trained and evidenced to be competent, and who participate in recognised ongoing continuing medical education, continuing professional development, and external quality assessment schemes
- describe the level of training required for staff delivering all aspects of the programme
- specify agreements to submit data as required, to allow for monitoring and operate within a framework of relevant data sharing permissions to enable pooled analyses to inform further design improvement
- facilitate quality assurance (QA) and audit activities
- follow QA advice to improve the service
- use the agreed common data standards and definitions
- describe how smoking cessation is integrated into the programme
- facilitate research studies into lung cancer early detection and screening, prevention and early detection of lung cancer and other significant pathology (including non-pulmonary morbidity). This may include enabling sharing of data and biological specimens when undertaken in accordance with research governance and data protection principles
- facilitate development of new knowledge and learning or processes to improve the programme.

1.4 Capacity and infrastructure

1.4.1 There should be sufficient capacity and infrastructure to deliver the programme, including:

- appropriate and suitable facilities for siting of mobile CT scanners, where required
- appropriate and suitable facilities for supporting assessments for eligibility and health checks
- scanning capacity
- appropriately trained radiographers
- appropriately trained radiologists

- clinical service for work up of referred participants including pathology and lab services.
- clinical service for treatment of participants
- smoking cessation support and very brief advice (VBA)
- administrative support for the programme including appointment scheduling, data collection, collation, analysis and submission
- data collection, that should be performed through a data management system to ensure efficient and secure data collection, communication and participant management. Data management systems should be comprehensive with built in failsafes, including invitation, reminders, scheduling, outcomes and follow-up and record data that enable future research and innovation. systems in place to regularly review requirements to adjust capacity and infrastructure as dictated by demand.

1.4.2 Provision of services should take place in the community where this improves participation or participant experience cost-effectively.

1.4.3 The implementation of the programme should be aligned with local services. This will involve working with regional and local healthcare management including:

- NHS England regional offices
- cancer alliances
- integrated care boards and integrated care systems
- local NHS trusts
- primary care networks and local medical committees
- imaging networks
- community diagnostic centres
- local authorities
- local patient and public voice groups
- other screening programmes

2. Clinical governance

2.1 Clinical governance structure

2.1.1 Each programme will need to have robust clinical governance in place to ensure the effective delivery of care to people who are invited to participate. This section outlines the key clinical roles which are mandatory for each LCSP.

Figure 1: Targeted screening for lung cancer clinical governance structure

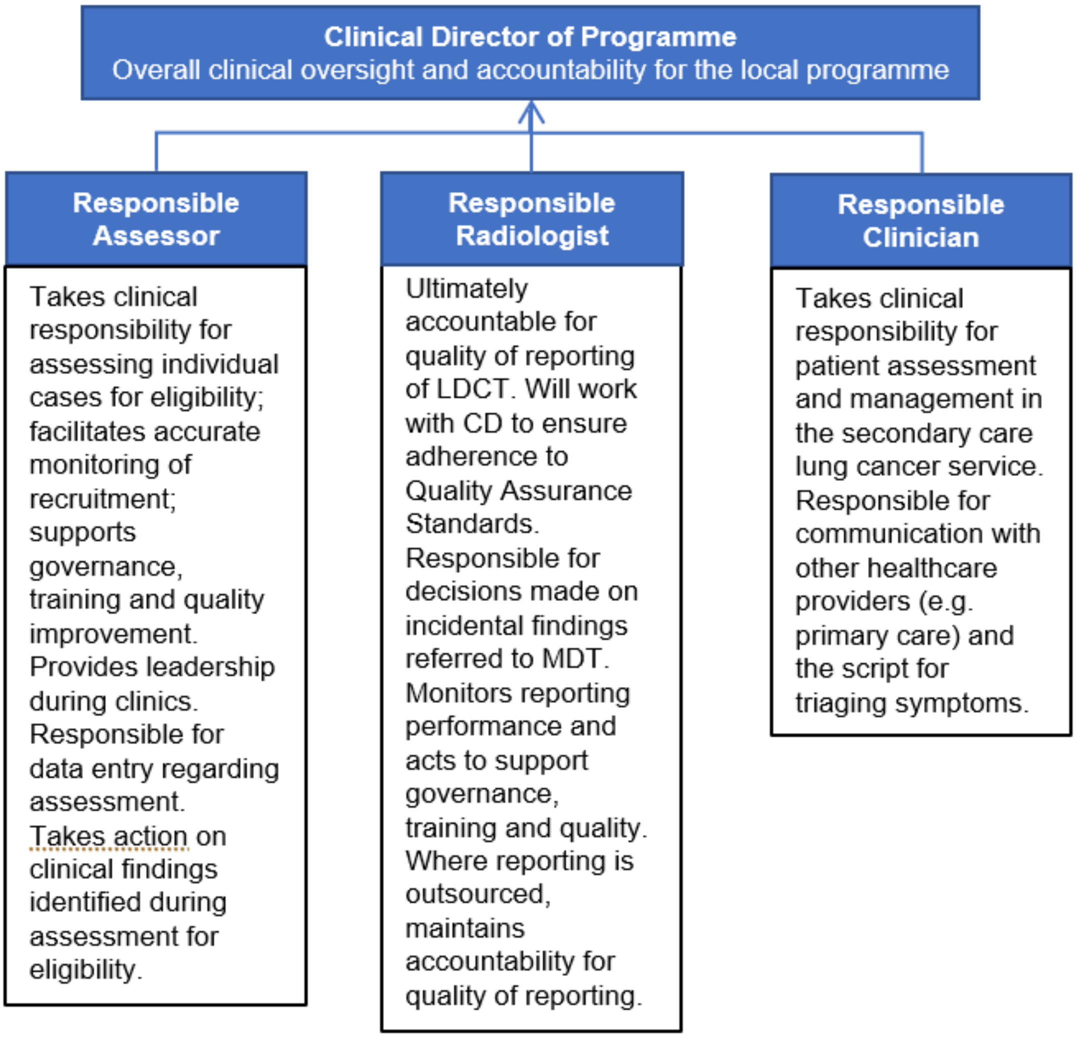

2.2 Description of key clinical roles

2.2.1 Clinical director of programme (CD)

2.2.2 There should be a single overarching clinical director role who has overall accountability for the QA Standards and safety of participants involved in the programme, including verifying the procedures for selection, scanning, acting on findings, resolving incidents and communicating with participants. These procedures should include failsafe mechanisms to ensure that decisions to recall participants for assessment are actioned, including reminders for individuals who fail to attend.

2.2.3 Responsible assessor (RA)

2.2.4 There should be a named clinician who is responsible for the leadership of the process to select and assess the individual cases for entry into the programme, the Lung Health Check (LHC) and any prior risk assessment for lung cancer.

2.2.5 The clinician can be a doctor, nurse or other professional with the appropriate clinical knowledge, authority and accountability, from either the local primary or secondary care team. They will continually oversee and monitor the clinical programme, the management of participants and provide day to day leadership of the clinical service. They will ensure:

- appropriate action is taken when clinical findings are identified as part of the assessment for eligibility; this may include further management in primary and/or secondary care
- clinical data and information are entered into the appropriate clinical system with a focus on data completeness
- that LHC risk assessment has appropriate QA processes.
- improvements and corrective actions are implemented to support governance, training and improve quality.

2.2.6 Responsible radiologist (RR)

There should be a named radiologist who is ultimately responsible for the justification of exposures and reporting of LDCT for the site. This will normally be a first-read radiologist who oversees all other radiologists reporting. They will work with the CD to ensure adherence to QA standards, and usually lead the radiology component of the Screening Review Meeting (SRM). They will be responsible for decisions made

regarding incidental findings referred to SRM. They will monitor reporting performance, and act on these results to support governance, training and quality. Where radiology reporting is outsourced, the RR retains accountability for overall quality of reporting within the site, in collaboration with the outsourcing partner.

2.2.7 Responsible clinician (RC)

There should be a named secondary care respiratory physician who is responsible for managing the referrals into the secondary care lung cancer service and coordinating the clinical assessment and management of participants in secondary care. They are responsible for approving all communications with other healthcare providers (e.g. primary care) and the participant, they are also responsible for the script for triaging symptoms (see 3.1.11). This will normally be the respiratory physician who works in the lung cancer service and who receives referrals from the programme.

2.3 Responsibilities

2.3.1 The expected responsibilities of all roles should be followed as a minimum, ensuring governance is effective with a consistent approach across sites.

2.3.2 The clinical director role can be occupied simultaneously with any of the other responsible roles. Any responsible role can be job shared, however good governance requires that individual responsibilities should be clear to ensure accountability is understood and there are no service gaps.

2.3.3 Ideally, key clinical roles will be filled by staff employed by the local Trust but can be employed within the wider ICB. Key clinical roles should not be filled by employees primarily employed by external providers (outside of the NHS) providing services to the LCSP system the clinical role is responsible for. In some instances, for programme inception or resilience, it may be necessary to appoint clinical roles in breach of this, but this should only be on a short-term basis and with the permission of the national team. Clinical roles can be filled by NHSE staff where the majority of the individual’s time is spent directly employed by NHSE, but who also work for external providers in addition to their NHSE responsibilities.

2.3.4 Skills: Professionals involved in screening assessment are expected to fulfil the requirements for individual professional training and for their

continuing professional development. They should carry out assessments and procedures regularly, to maintain their skills and competence. They should meet the requirements of their regulatory body, where applicable, including supporting others in their own development.

2.3.5 Audit: The CD, working in collaboration with the responsible roles, is accountable for ensuring that the assessment process is appropriately carried out (RA); that reporting radiologists adhere to the protocols (RR); and clinical assessment and monitoring of participants is monitored (RC). This should be confirmed by audits of performance, including:

- number of assessments performed (RAs)
- quality of data entry and submission (RAs, RRs)
- quality of risk assessment, including percentage of cases where LDCT eligibility was adjusted following RA audit/review (RAs)
- quality of overall participant experience (RAs, RRs, RCs)
- adherence to details of this protocol (RAs, RRs and RCs).

2.3.6 National audit: The CD is responsible for ensuring that all data is available for inclusion in a national audit with the purpose of comparing the programmes and measuring the overall success and impact. Data submission will be according to a national minimum dataset and submission is mandatory.

2.3.7 Reporting: The CD reports to NHS England through the national LCSP team.

2.3.8 Steering group: The CD, RAs, RRs and RCs will normally come together through a programme steering group, chaired by the CD. Membership of the programme steering group should include representatives drawn from primary care, public health and patient advocates. There should be access to expertise relevant to the LHC (e.g. in smoking cessation, data collection etc.).

Table 1: Summary of key responsibilities

Responsibilities	CD	RA	RR	RC
Ensure the assessment process is appropriately carried out by all RAs	✓			

Adherence to details of the standard protocol and quality assurance standards		✓	✓	✓
Quality of data entry		✓	✓	✓
Ensure required data is available for inclusion in a national audit	✓		✓	✓
Report to NHS England through the Cancer Alliance Board	✓			

3. Assessment process

3.1 Initial invitation

3.1.1 Due to the poor quality of smoking record data, everyone aged 55-74 should be given the opportunity to participate in the LCSP by the time 100% national rollout of the programme is achieved irrespective of their existing recorded history of smoking. This is to ensure that no potentially eligible people are inadvertently missed but will mean more ineligible people will be contacted. Participants eligible for a Lung Health Check (LHC) are those who:

- are aged between 55 and 74 years, 364 days of age
- are registered with a GP practice in England
- have ever smoked.

Eligible participants will be assessed to calculate their individual risk of developing lung cancer.

3.1.2 Participants invited should proceed through the pathway, even if the LHC or baseline scan would take place after the participant turns 75. However, no participant should receive a LHC or baseline scan more than 3 months after turning 75. Invited participants over the age of 75 should exit the programme in line with Section 7.3.

3.1.3 If a participant declines an invitation to a lung health check or does not attend, they should be re-invited according to local processes. Participants should be re-invited in the next screening round unless explicitly stating a wish not to be re-invited. If participants have suggested they no longer wish to be invited, the opportunity should remain for them to opt back in to the programme (See 7.1.5).

3.1.4 Participants who have smoked fewer than 100 cigarettes over their life are certain to not reach the risk threshold for CT and can be considered as low-risk.

3.1.5 GP and/or site records should be re-queried at least every two years from the initial query, to determine who to invite/re-invite (see section 3.3.18). This timescale will reduce in future iterations of the Standard Protocol, as the programme approaches full rollout.

3.1.6 GP re-query should take into account identification of participants previously ineligible by age.

3.1.7 Invitation to attend a Lung Health Check may be by written correspondence, telephone and text.

3.1.8 Initial assessment for suitability for a LHC and the actual LHC appointment must at least be offered via telephone or video-call. The gold standard is that participants should be able to choose whether the LHC is conducted remotely or face-to-face.

3.1.9 Initial assessment can be conducted by a Band 3 member of staff, but a Band 4 is recommended, ahead of a Band 6 (or greater) specialist nurse conducting the LHC. Initial assessment, or pre-population of risk calculator data is distinct from the lung health check clinical assessment (see section 3.3).

3.1.10 When initial assessment results in a non-clinical assessor excluding participants from LHC assessment, the lower of 50 or 1% of such cases per quarter should be audited by the RA or a delegated clinician not junior to a Band 6 LHC specialist nurse, for example by review of recorded telephone consultations.

3.1.11 Each participant’s risk score, components of medical history used to determine risk scores, and whether the participant was eligible for LDCT or not, must be clearly recorded. Where a participant is eligible for LDCT scan but excluded, the reason(s) for exclusion should also be clearly recorded.

3.1.12 Individuals will be assessed by confirming medical, social and employment history and risk factors for lung cancer. Validated lung cancer risk assessment tools will be used to quantify risk (see section 3.3.5).

3.1.13 Where participants are referred to secondary care for investigation of potential lung cancer, they should be readmitted to the LCSP once any active secondary care treatment or monitoring has come to an end, with processes and failsafes in place to ensure this happens.

3.1.14 Where necessary, reasonable adaptations should be made to the approach to ensure the service is accessible to all, including those with physical and learning disability and mental illness (e.g. easy read documentation, or engaging a key worker in the invitation [1]).

3.1.15 NHS translation services should be available where required for individuals without adequate English as a first language (see 3.4).

3.2 Participant journey

3.2.1 Figure 2 illustrates the participant journey for both those assessed at the LHC as low risk of developing lung cancer and those at high risk. Appendix A provides a more detailed clinical pathway.

Figure 2: High-level participant journey

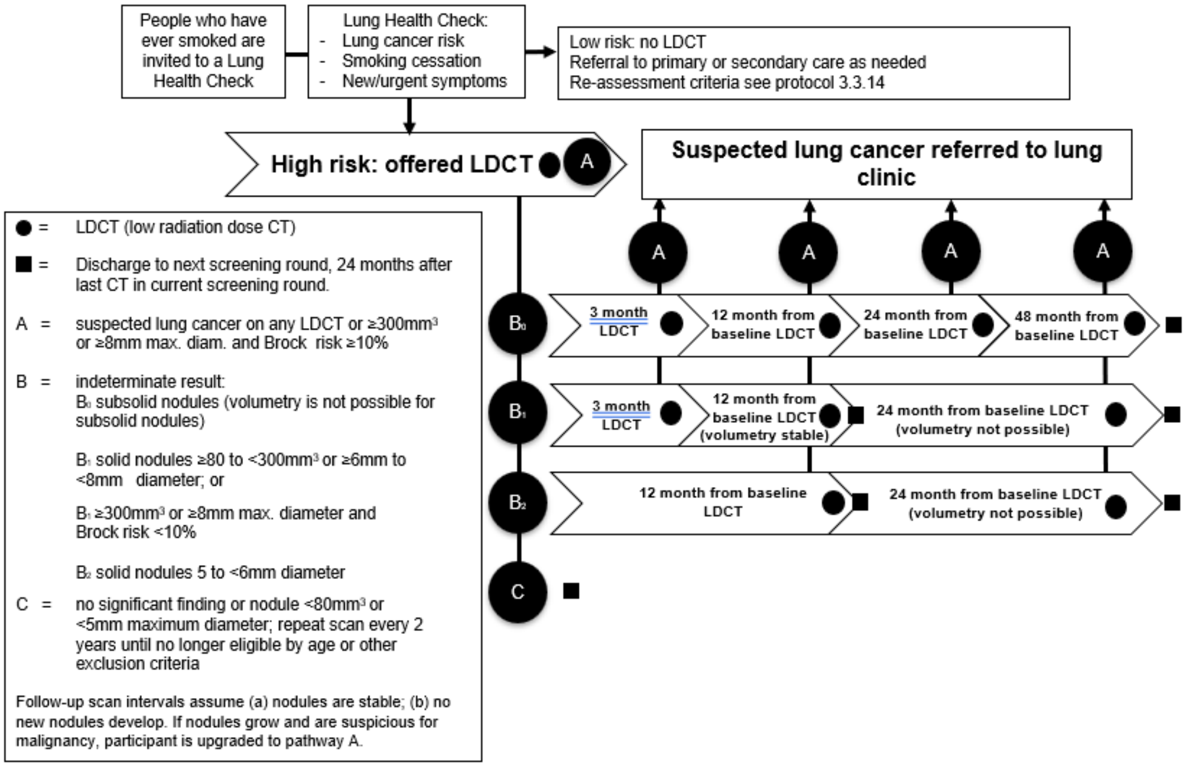

3.2.2 At the LHC or initial assessment, participants will have a discussion to assess their individual lung cancer risk. This will include questions about smoking habits. Those at low risk do not require a CT scan.

3.2.3 Current smokers will be offered smoking cessation Very Brief Advice (VBA) and formal smoking cessation service referral on an opt-out basis.

3.2.4 The initial assessment call must also query relevant medical history (in particular relevant exclusion criteria, see 3.3.12) and any new or urgent clinical symptoms that require further assessment (see section 3.6) with

escalation as appropriate to clinical colleagues with onward referral to primary or secondary care as needed.

3.2.4.1 Clinical assessment should not be undertaken by non-clinical staff – patients who describe symptoms on the initial assessment should always be referred to clinical staff.

3.2.4.2 Where an initial assessment step is included as part of the risk assessment process, the RA and RC must put in place a robust process to make sure participants with symptoms are assessed by a clinician – urgently where appropriate.

3.2.4.3 An appropriate script approved by the Responsible Clinician for assessing symptoms must be in place, with escalation to a clinician (e.g. an LHC nurse) if any urgent symptoms are identified. It may be appropriate to do so by telephone in advance of the LDCT scan and a clinician should be available for this purpose.

3.2.5 Any participant assessed as being at high risk of lung cancer will have their proposed exposure to LDCT justified and authorised in line with IR(ME)R regulations and then be offered a low-dose CT scan within 56 days of the risk assessment, if there are no exclusion criteria. The scan will typically show one of three things:

i) No significant findings or nodules with a volume of $<80mm^3$ or 5mm in maximum diameter.
ii) Indeterminate results.
iii) Something that requires further investigation.

Results	Action
No significant findings or nodules	Return at next screening round in 24 monthly intervals from the last stable or normal scan.
Indeterminate result	Further surveillance scans at three and/or 12 months from baseline, and where appropriate further scan at 24

	months from their baseline scan (see Figure 2); then return to next screening round assuming all findings stable or no significant change*.
Requires further investigation	Referred to local specialist lung clinic

*No significant growth or altered morphology/consistency

3.2.6 Table 4 includes a more detailed nodule management protocol.

3.2.7 Surveillance scans (3months/12months) should be completed a maximum of 28 days after the target date but should not be completed earlier than the target date.

3.2.8 Screening round scans (24 months) can be completed up to 56 days before or after the target date.

3.2.9 Participants returning for surveillance scans or screening round scans should not receive a further Lung Health Check but should be checked for continuing eligibility for LDCT. Participants that did not previously breach the risk threshold, who have been reinvited, should receive a further Lung Health Check.

3.2.10 Participants with non-cancer related symptoms will be referred to their GP or appropriate specialist if required.

3.2.11 Where participants are referred to secondary care for investigation of potential lung cancer, they should be readmitted to the LCSP once any active secondary care treatment or monitoring has come to an end (See 3.1.13).

3.3 Risk assessment

3.3.1 Assessment of risk of lung cancer is essential to maximise the cost effectiveness of the intervention. There are a number of methods, and further research may lead to newer models in the future. This will form part of the evaluation of the THLC programme. Initial assessment, or pre-population of risk calculator data (see section 3.1.11) can be performed by

appropriately trained admin staff. It is recommended these staff are at least Band 4, but at a minimum must be Band 3. The decision to refer to LDCT requires review by an IR(ME)R trained doctor or nurse of Band 6 or greater with experience of conducting lung health checks. A representative sample of cases excluded from LHC assessment by a non-clinician should be audited by the responsible assessor, or a delegated clinician not junior to a Band 6 lung health check nurse. Where the assessment is undertaken by an external provider, the provider can support this audit / QA process but the RA (or where this is also contracted to the external provider), a member of the Screening Review Meeting (SRM) must oversee the audit and directly assess the lower of 50 or 1% of cases per quarter (Call recording of pre-assessment steps may be useful in this scenario.

3.3.2 Where initial assessment or LHC has taken place over the phone or virtually the participant's self-reported height and weight can be used, but every effort should be made to ensure that this is accurate.

3.3.3 The LCSP will use the prostate lung colorectal and ovarian $(PLCO)_{M2012}$ risk prediction model and the Liverpool lung project (LLP) version 2 [2, 3] to select participants to be offered an LDCT. The American $PLCO_{M2012}$ model has been adapted for use in the UK to reflect UK ethnic groups.

3.3.4 Evidence suggests that a risk threshold of ≥1.51% risk of lung cancer over six years is the minimum threshold for $PLCO_{M2012}$, and ≥2.00% risk of lung cancer over five years for LLPv2 [4, 5]. However, the latter has only been shown in modelling studies and may lead to substantially more LDCTs. Thus, a risk threshold for LLP of ≥2.5% is considered the minimum threshold.

3.3.5 ~~$_{M2012}$~~The factors used in these models that would need to be collected are shown in table 2 below:

Table 2: Factors included in two multivariable risk prediction models

LLPv2: ≥2.5% risk	$PLCO_{M2012}$: ≥1.51% risk
• Age<br>• Sex<br>• Smoking duration (years)<br>• Previous pneumonia/ COPD/ emphysema/ bronchitis/ TB<br>• Occupational asbestos exposure<br>• Previous history of malignancy<br>• Previous family history of lung cancer<br>– relative's age at onset, ie <60 years or >60 years<br>– whether first degree relative.	• Age (years)<br>• Education level<br>• Body mass index<br>• COPD/ chronic bronchitis/ emphysema<br>• Personal history of cancer<br>• Family history of lung cancer<br>• Ethnicity*<br>• Smoking status<br>• Average number of cigarettes smoked per day<br>• Duration smoked (years)<br>• Years having ceased smoking.

* referred to as 'race' in the original $PLCO_{M2012}$ risk model. Modifications for UK ethnicity have been made.

3.3.6 For the purposes of the LCSP, participants satisfying either LLPv2 or $PLCO_{M2012}$ are to be considered eligible for a low-dose CT provided they meet the inclusion criteria in 3.3.10 and do not have any of the exclusion criteria listed in 3.3.8.

3.3.7 Inclusion criteria:

- Age is from 55 years to 74 years and 364 days.
- Willing and able to undergo LDCT.
- $PLCO_{M2012}$ risk of ≥1.51% over six years or LLPver2 five-year risk of ≥2.5%.

3.3.8 Exclusion criteria:

- Weight or physical size exceeds restrictions for scanner (e.g. >200kg).
- Participant unable to lie flat (every effort should be made to support the participant to comfortably lie on the scanner bed before exclusion).
- Poor physical fitness such that treatment with curative intent would be contra-indicated. This may require a second opinion or advice from the local lung cancer MDT. This may include:
    - Participants registered on the palliative care register

- Participants with severe frailty (electronic frailty index >0.36)
- Participants with metastatic cancer but not metastatic prostate cancer, metastatic breast cancer or metastatic melanoma (unless participants are already receiving annual diagnostic chest CT scans).
- Participants with mesothelioma
- Participants currently under active treatment for lung cancer or under surveillance for lung cancer following treatment

3.3.9 Participants who have had a full thoracic CT scan that meets the image reconstruction parameters of the programme (see section 4.4.) in the last 12 months are not excluded from the programme, but would have their LDCT appointment deferred until 12 months have elapsed since that last scan, provided they:

- still meet all inclusion criteria
- have no exclusion criteria

3.3.10 Participants who lack the mental capacity to consent to screening should not be excluded from the programme. ‘Consent to cancer screening’ guidance on informed consent should be followed, including the use of best interests decisions.

3.3.11 If a patient is unable to mount the scanner, then every effort should be made to allow them to be scanned.

3.3.12 Reasonable steps should be taken to assess whether a full thoracic CT scan has taken place and defer the LDCT appointment if appropriate. However, in the absence of any conclusive evidence of a prior full thoracic CT scan, the participant should be given benefit of doubt and booked for an LDCT. If a participant receives an out-of-programme (OOP) CT scan covering the thorax between LCSP scans (either screening round or surveillance scans), the date of the participant’s planned LCSP scan should not usually be deferred. SRM can defer scans if a clinical role believes SRM should consider deferral. The fact that the participant has had an OOP scan should be flagged on the participant’s record on the LCSP database. If there is a new nodule or finding on the planned LCSP scan since the last LCSP LDCT, review the case with the OOP CT scan at the Screening Review Meeting.

3.3.13 Effort should be made to check continuing eligibility for LDCT in advance of surveillance or screening round scans.

3.3.14 Where participants are assessed at below the threshold for LDCT, where possible they should be reinvited at the point where they are likely to become high risk. Previously ineligible participants must receive a repeat LHC ahead of any LDCT – this LHC could be informed by historical data but this should be carefully verified with the participant.

3.4 Information for participants

3.4.1 Where available, national participant information resources, developed by appropriate patient and public communication experts and co-designed with diverse representatives of the lung screening-eligible population, should be used. Where necessary, for example to improve equity, diversity and inclusion, sites can include minor changes, that should be limited to pre-specified sections of text, with the support of the NHS England team.

3.4.2 All communications should be written in sufficiently lay language to address barriers to participation and support informed decision-making through an understanding of the risks and benefits of lung screening.

3.4.3 Written and/or video information should be provided at all stages, with specific information on what is involved. For those eligible for LDCT, this should include the risks and benefits of the test. This should be followed by a discussion between the individual and the clinician to facilitate informed decision-making and subsequent acceptance/decline of the test.

- The focus should be on informed choice and supporting participant understanding of risks associated with taking part in lung cancer screening, for example using lay explanations, infographics and easy read versions.
- It should be made clear what the screening programme can find and what it cannot. Specifically, it should be made clear that the LDCT is designed to detect lung cancer, the test is not optimised to find anything apart from lung cancer, but incidental findings are possible.
- Information should be available at all relevant points throughout the pathway.
- A trained interpreter should be available during appointments where the functional language is not English.

- Participants with learning disabilities should be provided with appropriate support to enable them to understand all processes and results.
- All information will be provided in accessible font sizes and in plain English.
- Flexible appointments and all reasonable adjustments will be made for screening participants with physical or learning disabilities or impairments.
- Optimal communications should be co-designed and evaluated with representatives of the intended audience.

3.4.4 As part of an LHC or initial assessment, all current smokers must receive very brief advice on smoking cessation by a trained professional and be referred to smoking cessation services on an opt-out basis.

3.4.4.1 Smoking cessation advice and information about locally available support must be incorporated into written correspondence and should be face-to-face where possible. Enhanced smoking cessation interventions are also encouraged including the use of pharmacotherapy.

3.5 Consent process

3.5.1 Consent for CT screening should be taken by a suitably trained clinician or non- clinician, familiar with the risks and benefits of the process and radiation exposure. Participants should be informed of the following:

- The primary purpose for undergoing LDCT is to identify lung cancer at a stage when there may be options for curative treatment. Information about the estimated chance of finding a lung cancer should be stated in accessible language.
- If lung cancer is identified, the participant will be directly referred to an appropriate lung cancer service and managed according to the National Optimal Lung Cancer Pathway.
    - The LDCT scan technique is not optimised to detect abnormalities other than lung cancer, but such respiratory and non-respiratory abnormalities can sometimes be detected by LCSP. If potentially significant conditions are identified that require action, the participant will be informed and the LCSP will either make an appropriate referral and/or advise the participant's GP on the appropriate action to be taken.

The participant and GP will not be informed of any clinically non-significant findings for which there is no recommended treatment. Action on incidentally detected abnormalities should follow the LCSP Incidental Findings Protocol.

- Indeterminate pulmonary nodules are often benign.
- LDCT uses low radiation with information about the associated risks.
- A negative CT scan does not exclude the possibility of having lung cancer in the future. Participants should be informed about the need to report future symptoms of lung cancer if they develop.
- Cancer may be identified that would not have led to harm (over diagnosis).
- There are some risks of harm relating to the further investigation and treatment of findings on the CT.
- Protocols will be followed that minimise harms from further investigation and false positives.
- Participants will be asked to consent to the retention of clinical data and radiological images for evaluation and future research purposes, under the correct NHS and Human Research Authority (HRA) governance procedures. It should be made clear to the participant that:
    - such data enables improvements to the LCSP process and quality control. Data will usually be anonymised unless it is considered necessary to link lung health check records to other health records.
    - not providing this level of consent, or declining to participate in a specific research programme, does not stop them in any way from taking part in this programme.

3.5.2 It is recommended that consent to data usage, also includes consent to approach by research teams in future.

3.5.3 Either verbal or written consent are acceptable.

3.5.4 As a minimum the clinical record must indicate that the participant has been informed of the benefits and risks of LCSP LDCT and gives consent to proceed. A separate statement/entry in the medical record should be made that the participant consents to research data use ± contact by the research team.

3.5.5 Details of what consent was given and how this was elicited should also be recorded in the clinical record.

3.6 Pathways for new symptoms

3.6.1 Participants at high risk of lung cancer often have comorbidities that cause symptoms. These may be unrelated to cancer and – in the circumstances described below in section 3.6.5 – permit continuing with the LDCT screen.

3.6.2 Any new or urgent clinical symptoms that require further assessment should be escalated as appropriate to clinical colleagues with onward referral to primary or secondary care as needed.

3.6.3 Those presenting with respiratory infection should be booked in for a deferred appointment in six weeks' time, to avoid false positive results. Evidence of respiratory infection will be assessed at time of appointment, including cough, new or changed sputum colour or volume, breathlessness, wheeze, chest pain, fever, sore throat and coryza.

3.6.4 If the individual presents with clinical features of lung cancer or any of the following symptoms they should proceed directly to an urgent CT of the neck, thorax and abdomen with administration of intravenous contrast.:

- persistent haemoptysis
- signs of superior vena cava obstruction (SVCO) such as face and/or neck and/or arm swelling, raised and non-pulsatile jugular venous pressure (JVP)
- stridor
- signs of malignant cord compression (new onset back/shoulder pain, sensory and/or motor deficit, urinary and/or faecal incontinence, gait abnormalities).

3.6.5 If potential participants present with symptoms consistent with exacerbation of COPD or other chronic pulmonary conditions, they should proceed with the LDCT.

3.6.6 Participants who meet eligibility criteria for a LDCT but who have any of the following features or symptoms, as described in NICE referral criteria (NG12), should have their LDCT expedited and prioritised for reporting:

- cough
- fatigue

- dyspnoea
- chest pain
- unexplained weight loss
- appetite loss
- persistent or recurrent chest infection
- finger clubbing
- supraclavicular lymphadenopathy or persistent cervical lymphadenopathy
- chest signs consistent with lung cancer
- thrombocytosis.

3.6.7 Those who are ineligible for LDCT due to a low-risk score should be managed according to the NICE NG12 cancer recognition and referral guidelines. Local arrangements for requesting urgent chest X-rays and direct referral for CT may reduce delays.

3.6.8 If a participant is not physically well enough to receive a CT scan then this should be deferred.

3.7 Lung health check personnel training

3.7.1 LHC personnel should be trained in all necessary procedures, for example:

- Informed consent (national consent training)
- Lung cancer risk assessment (including LLP and PLCO scores)
- Employer training for entitlement to refer under LCSP (IR(ME)R compliant CT referral for anyone requesting a scan)
- Very Brief Advice on smoking cessation
- Process for onward referral.

4. Low dose computed tomography acquisition and reading

4.1 CT equipment and volumetry software requirements

4.1.1 The minimum specification is for a 16-channel multi-detector CT, fixed site or mobile, calibrated and appropriately acceptance tested. According to the manufacturer's specifications, capable of delivering low radiation dose protocols. Most modern scanners exceed this specification and will achieve this.

4.1.2 Volumetry should be used for assessment of solid pulmonary nodules as per British Thoracic Society recommendations (REF) (see section 4.7).

4.1.3 Volumetric segmentation of the nodule should be visually assessed for reliability.

4.1.4 The volumetric software should ensure display of all volume metrics, including percentage volume change and volume doubling time (VDT) when more than one LDCT is available.

4.1.5 VDT must be calculated from the first appearance of a nodule [e.g. for a nodule detected at the first (baseline) LDCT, VDT should be calculated from that baseline LDCT.

4.1.6 Software and technique should remain consistent to allow accurate comparison of volumes. When the supplier of volumetric software performs software updates/upgrades, the dates of such upgrades should be recorded and a log of version control kept. The supplier should provide evidence that the upgrade provides the same measurements or ensure that the user is prompted to re-measure nodules from preceding scans if the software upgrade alters measurements.

4.1.7 Volumetric software must be directly or indirectly integrated into picture archiving and communications systems (PACS), capable of automated image retrieval of, and comparison with, historical imaging where appropriate.

4.1.8 Other desirable features are high automated segmentation accuracy rates (>85%), and automated structured reporting.

4.1.9 Computer aided detection (CAD) systems should be used in a concurrent or second reader format. A false positive rate of <2 per case is required for CAD systems.

4.2 CT image acquisition protocol

4.2.1 Subject position: Participants should lie supine on the CT table with arms above their head and thorax in the isocentre of the scanner. Subject comfort should be optimised, and maximal inspiration rehearsed prior to the scan to minimise motion during the CT. Imaging should be performed during suspended maximal inspiration. No intravenous contrast material will be administered.

4.2.2 Localiser: Sites should use their standard scout view (scanogram) to localise the start and end positions of the scan. The frontal localiser should be performed in the PA projection and at the lowest possible setting to minimise breast dose.

4.2.3 Volumetric CT scan: The lung parenchyma (lung apices to bases) must be scanned in its entirety in a single cranio-caudal acquisition. The field of view selected as the smallest diameter as measured from widest point of outer rib to outer rib large enough to accommodate the entire lung parenchyma.

4.2.4 Participant height and weight should be recorded alongside other participant data entered as part of the CT scan.

4.3 Exposure factors

4.3.1 Radiation exposures will be as low as possible while maintaining good image quality. The acquisition parameters will be set to ensure that the calculated radiation dose delivered to each individual is below 2 mSv (based on the median standard 70kg adult). This will be done by ensuring that the kVp and mAs settings are varied according to participant body habitus.

4.3.2 The height and weight of participants will be used to enable accurate selection of exposure factors. Ultra LDCT should be used where available and considered to be of equivalent diagnostic sensitivity to LDCT.

4.3.3 Appropriate standardisation of image quality to ensure reproducible high image quality should be performed.

4.3.4 Medical physics input should be obtained in advance of setting up LDCT scanning protocols.

4.3.5 The cause of poor diagnostic quality images should be documented where possible.

4.4 Image reconstruction

4.4.1 Image reconstruction should be standardised and used for any subsequent follow-up examinations where possible, with particular emphasis on ensuring that slice thickness, reconstruction increment, and reconstruction algorithm are identical.

4.4.2 Slice thickness must be ≤ 1.25mm. An example of reconstruction parameters used in low-dose screening CT are outlined in table 3 below.

4.4.3 If iterative reconstruction is used, this should be kept consistent at follow up.

Table 3: Example of reconstruction parameters for LDCT

Reconstruction algorithm	Reconstruction thickness	Reconstruction increment	Reconstruction FOV
Moderate spatial frequency/soft tissue	1mm	0.7mm	Entire lung parenchyma

4.5 Image interpretation

4.5.1 Image interpretation should be performed on systems which permit scrolling through the data set with variable thickness and orientation using multi-planar reformations (MPR) and maximum intensity projection. Where volumetry is used, radiologists should visually check for appropriate segmentation of nodules.

4.5.2 All reconstructed scan data (according to minimum requirement for volumetric analysis) acquired from the participants should be archived and retained at a local or central site.

4.5.3 There should be a mechanism for obtaining the previous thoracic CT imaging for the participant, as far as is practically possible (see also 3.3.15 to 3.3.17).

4.6 Thoracic CT reader

4.6.1 Lung cancer screening CT reading requires unique skills beyond the skillset needed for clinical thoracic CT reading. Lung cancer screening requirements include the following:

- Radiologists should report a minimum of 500 thoracic CTs per annum in their routine clinical practice, the majority of which should be CTs performed for the evaluation of lung cancer (this should include a mix of LCSP LDCTs, pulmonary nodule follow-up and symptomatic lung cancer).
- Radiologists should participate in a thoracic MDT meeting as part of their routine clinical work. This excludes Screening Review Meeting (SRM) attendance, as those are problem-solving meetings for lung cancer screening and not for lung cancer diagnosis and management.
- Readers must be familiar with the use and limitations of nodule volumetry software and apply the BTS guidelines for nodule management in their usual practice.
- Radiologists should complete a Royal College of Radiologists-British Society of Thoracic Imaging (RCR/BSTI) training course approved by NHS England on CT screening.
- Radiologists will participate in regular personalised performance review via a centrally curated educational self-assessment and training scheme for LHC CT reading (PERFECTS), as described in the Quality Assurance Standards
- The RR is responsible for ensuring all the above criteria are met, and for all relevant aspects of clinical governance.

4.6.2 The quality of the scan should be documented as diagnostic or non-diagnostic in the CT report. If non-diagnostic, the reasons should be given. Protocols should be in place for efficient recall of these participants once the reasons are understood.

4.6.3 Reading workload and environment

- Radiologists should aim to report 12-20 scans per PA of clinical time, with a view to achieving 20 or more scans per PA as screening reporting experience and familiarity with automated reporting increases.
- The reporting environment must meet minimum standards for ergonomics including screen and chair position, room lighting and display requirements set out in the RCR recommendations; this should also apply in home reporting situations.

4.6.4 The lung nodule size threshold for characterisation is ≥ 5mm or $80mm^3$. Where multiple nodules are detected, at least two nodules, including the largest nodule, and where possible all nodules $>200mm^3$, should be recorded. Smaller nodules may be characterised for research purposes. All new nodules on interval LDCT $\geq 30mm^3$ or ≥4mm max diameter, and that are not thought to be due to infection/inflammation, should be reported as this determines scan interval in these nodules (see Table 4).

4.6.5 Programmes should follow national guidelines and have protocols in place for reporting and management of incidental findings (see section 6.4). Narrative/descriptive reports should be avoided. Clinically non-significant findings should either not be reported or clearly identified as such. An emphasis should be placed on reporting of findings where there are proven interventions for participant benefit.

4.7 Volumetric analysis

4.7.1 Solid nodules should be measured using semi-automated volumetry. Where volumetry segmentation is not possible or judged to be inaccurate, maximal axial manual diameter measurements should be recorded on lung window settings, excluding any spiculation. Manual adjustment of volumetric analysis should be avoided as this may introduce unquantified variability.

4.7.2 Subsequent scans should measure volume in the same solid nodules and a volume doubling time (VDT) calculated for each where an increase ≥25% has occurred (as a <25% difference in volume may be within the limits of expected inter-scan variation). A less than 25% increase may be within the margin of error. Where volumetry is not possible, the growth rate should be based on visual assessment or diameter measurements, accepting that this can be less accurate.

4.7.2.1 Where more than one prior scan is available, VDT should be calculated relative to the earliest (preferably baseline) LCSP LDCT with technically acceptable segmentation.

4.7.3 Images showing the boundaries of solid nodule volume segmentation, including size and VDT calculation where appropriate, should be available to radiologists to allow visual assessment of segmentation reliability. This assists with the reading process at follow up and ensures that the information is efficiently conveyed to the lung cancer MDT or Screening Review Meeting for relevant cases.

5. Surveillance of indeterminate nodules (pathway B)

5.1 Scan intervals

5.1.1 Nodule management should be protocolised and based upon the BTS 2015 pulmonary nodule guidelines [6] and NICE guidelines for the management of lung cancer [7].

5.1.2 Local or regional modification of nodule management guidelines is not encouraged. Where such modification is deemed necessary, clinical justification should be provided and permission for modification sought.

5.1.3 Participants with CT scans showing nodules are managed according to nodule composition and size. Volumetry is the preferred method for solid nodules.

5.1.4 Maximum axial diameter is used in the case of:

- solid nodules with unreliable segmentation
- subsolid. (i.e. part-solid [PSN] and ground glass [GGN] nodules: Management is dependent on the overall maximum diameter of the nodule, and of the solid component in case of PSNs.)

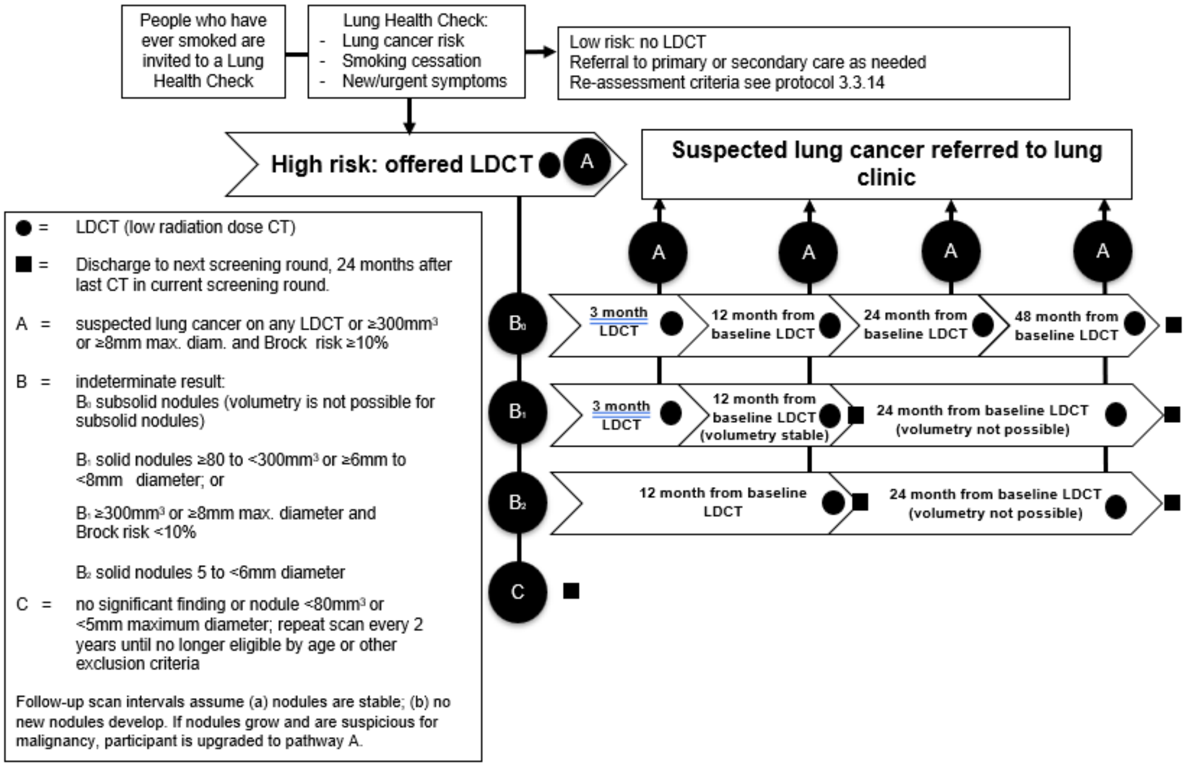

5.1.5 Table 4 below shows how the nodule size affects follow-up interval and referral:

Table 4: Nodule size and follow-up interval and referral (*must be interpreted in conjunction with notes below*)

Notes and definitions:

- **“Stable”**: as per current guidelines
    - Solid nodules with reliable volumetric segmentation: percentage volume change (relative to the nodule’s first occurrence) of <25%
    - Solid nodules with unreliable segmentation: difference in maximum diameter of <2mm
    - Sub-solid nodules: difference in maximum diameter of <2mm of the entire nodule or the solid component if present
- Baseline screening round: the first screening round that the individual undergoes, which includes their baseline (first) scan in the programme and all follow-up CT scans performed during that screening round. This timepoint is often referred to as T0. The screening round concludes when the minimum follow-up period for any indeterminate findings concludes and there are no new findings, or when the participant is referred to a lung cancer service, whichever is sooner.
- Incident screening round: screening rounds that occur after the baseline round. These timepoints are often referred to as T1, T2, and so on.

5.1.6 Next screening round: 24 months after the last stable or negative scan. E.g. if a solid nodule with volume ≥80 to <300mm^3 at baseline CT is stable after 12 months of follow-up, the next screening round is 24 months from that point, i.e. 36 months from baseline.

5.1.7

- Size thresholds change where nodules were not seen on a previous CT. Follow-up is based on the same minimum time-periods as for nodules seen at baseline: at least 12 months for a reliably volumetrically segmented nodule, and at least 24 months for a non-volumetrically segmented nodule.
- This table provides recommendations for surveillance scans **relative to the current scan**. For example, ‘follow-up in another 9 months on a 3 month scan is the same as ’12 months from baseline’

- In the event a participant has a delayed follow-up scan, radiologic judgement should be applied to decide when the subsequent follow-up scan (if still needed) should be performed, with the principle at all times being to bring the participant back in line with the original timeline for their follow-up, while at the same time allowing sufficient time to pass to enable reliable estimation of stability or growth. For example, following a baseline scan, if a 3-month scan to follow up a volumetrically segmentable indeterminate solid nodule only occurs at 7 months from the baseline scan, if the nodule is stable there is no point in performing another scan 3 months from then (i.e. 10 months from the first scan); it would be sensible to choose the next best option (e.g. 6 months from the current scan which would be a total of 13 months from baseline- and still within 1 month of the original intended 12-month follow-up from baseline). Where there is any doubt, discussion at the SRM is encouraged to ensure consistency.
- Radiologists should use their clinical discretion to dismiss overtly inflammatory nodules, especially ground-glass nodules, even if these are flagged by the computer-aided detection system. These systems are only meant to be aids to reading and the radiologist decides the final disposition of any nodule. NB: Please note the Incidental Findings guidance on consolidation- if Inflammation more likely than cancer refer to SRM consider repeat CT Repeat CT at 6 weeks or 3 months depending on concern (within or outside screening programme).
- The timepoint of the scan being reported should be explicitly made clear to the radiologist at the time of reporting.
- For new nodules, we emphasise only discrete nodules considered non-inflammatory should be designated for follow-up.

CT Nodule type and size (measure)	Follow-up interval from current scan
**BASELINE ROUND CT**	
No nodules	None- discharge to next screening round
**Solid nodules**	
<80mm^3 or <5mm max. diam.	None- discharge to next screening round (NB: if a prior adequate non-screening thoracic CT is available, and the nodule is proven to be new in comparison, the lower threshold for new solid nodules apply – see below)
≥80 to <300mm^3	3 months, if stable in another 9 months, then discharge to next screening round if stable
≥6mm and <8mm max. diam. (volumetry not possible)	3 months, if stable in another 9 months, then a further 12 months, then discharge to next screening round if stable
5 to 6mm max. diam. (volumetry not possible)	12 months, if stable in another 12 months, then discharge to next screening round if stable
≥300mm^3 max. diam. And Brock risk <10%	3 months, if stable in another 9 months, then discharge to next screening round if stable
≥8mm max. diam. And Brock risk <10% (volumetry not possible)	3 months, if stable in another 9 months, then a further 12 months, then discharge to next screening round if stable
≥300mm^3 or ≥8mm max. diam. And Brock risk ≥10%	Refer
**Sub-solid nodules**	
<5mm max. diam (including PSNs with solid component <5mm max. diam)	None- discharge to next screening round
Ground-glass nodule ≥5mm max. diam.	3 months- if persists assess malignancy risk (size, morphology, density, Brock score)- if low risk in another 9 months, then 12 months, then

	24, then 48 months. If stable, discharge to the next screening round
Part-solid nodule, solid component ≥5mm max. diam	3 months- if persists assess malignancy risk (size and growth of solid component, morphology, density, Brock score)- in another 9 months, then 12 months, then 24, then 48 months. If stable, discharge to the next screening round
**Pericystic lesions***	In the absence of formal guidance on these types of lesions in the current BTS guidelines, follow-up based on the presence and characteristics of any associated ground-glass or solid component, as appropriate
**INCIDENT ROUND CT**	
**Solid nodules**	
Present on most recent prior CT	
Stable since the most recent prior CT	Discharge to next screening round if minimum surveillance period for nodule has been completed
Growing since the most recent prior CT	Consider referral for further investigation or CT surveillance depending on VDT and size of nodule (see section 6)
NEW discrete nodules since the most recent prior CT	
<$30mm^3$ or <4mm max. diam	None- discharge to next screening round
≥$30mm^3$, <$300mm^3$	3 months, if:<br>Resolves- discharge to next screening round<br>Persists but stable- in another 9 months, if stable discharge to next screening round
≥4mm, <8mm max. diam (volumetry not possible)	3 months, then if:<br>Resolves- discharge to next screening round

	Persists but stable- in another 9 months, then 12 months, if stable discharge to next screening round
≥300mm^3/≥8mm	Refer
**Subsolid nodules**	
Stable since the most recent prior CT	Discharge to next screening round if minimum surveillance period for nodule has been completed
Growing in size (in the case of part solid-nodules, size of solid component) or density, or altered morphology	Consider referral or closer CT surveillance depending on level of concern
NEW discrete nodules since the most recent prior CT	As per baseline round CT
**New discrete non-inflammatory nodules on any interval CT**	
At any timepoint: Solid <30mm^3 or <4mm max. diam. OR sub-solid <5mm max. diam.	None- continue with any planned screening or surveillance CT
**Solid**	
Appears at 3 month follow-up CT	3 months, if:<br>Resolves- discharge to next screening round<br>Persists but stable- in another 9 months, then 12 months, if all nodules stable discharge to next screening round
Appears at CTs performed after 9 or 12 months	
≥30mm^3, <300mm^3	3 months, if:<br>Resolves- discharge to next screening round<br>Persists but stable- in another 9 months, if all nodules stable discharge to next screening round

≥4mm, <8mm max. diam. (volumetry not possible)	3 months, if:<br>Resolves- discharge to next screening round<br>Persists but stable- in another 9 months, then 12 months, if all nodules stable discharge to next screening round
**Sub-solid**	As per baseline round CT

**Guidance on new pericystic lesions on incident round or follow-up CT is not given as a genuinely new pericystic lesion would be unlikely.*

5.2 Reducing measurement variability

5.2.1 Each CT scanner vendor has its own reconstruction algorithms. To reduce the impact of these, as much as possible the scanner make and model should be kept the same as the baseline.

5.2.2 If it is not practical to use the same make and model of scanner then efforts should be made to ensure the same vendor and reconstruction algorithm are used and the image quality and metrics remain comparable.

5.2.3 If it is not practical to use the same vendor then the same or similar reconstruction algorithm must be used. The Medical Physics Expert must be satisfied that the algorithms from different manufacturers have similar technical parameters.

5.2.4 Slice thickness and slice increment must be the same regardless of scanner make, model, vendor or reconstruction algorithm.

6. Pathway A: Findings requiring further investigation

6.1 Lung nodule management and follow-up/further diagnostics

6.1.1 The protocol for management of participants with significant findings should follow the BTS 2015 pulmonary nodule guidelines and NICE guidelines for the management of lung cancer. In brief:

- Nodules with confirmed VDT>600 days can be referred back for annual LDCT.
- Nodules with VDT 400-600days, surveillance or biopsy / resection can be offered depending on participant preference.
- Nodules with VDT<400 days should be further investigated, but only if they have reached a size suitable for meaningful intervention (e.g. PET-CT, percutaneous biopsy, lung resection, according to participant preference).
- For PSN, any change in morphology or growth of solid component (≥2mm) as well as a Brock risk of malignancy of >10% should prompt consideration of a histological diagnosis and definitive management. Such lesions have a better prognosis, so further observation may be indicated to avoid over diagnosis.
- For pure GGN, any change in morphology or appearance of solid component as well as a Brock risk of malignancy of >10% should prompt consideration of further imaging follow-up or histological diagnosis and definitive management, noting the very good prognosis of these lesions and potential for over diagnosis.

6.1.2 Nodules with a Herder risk score >10% following FDG-PET/CT may be referred and managed within the respiratory clinic or within the screening programme. Nodules with a higher score should be managed within the respiratory service. The Herder tool is validated risk calculator that incorporates findings from FDG-PET scans (available in BTS pulmonary nodule app).

6.2 Multidisciplinary team meetings

6.2.1 There are two multidisciplinary meetings that are relevant. All programmes should have access to these MDTs:

- The Screening Review Meeting (SRM), where the management of findings (either nodules or incidental findings) requiring urgent investigation, especially those with relevant prior imaging, are discussed and management plans are devised so that communication with the participant and any healthcare professionals can be co-ordinated.
- The lung cancer MDT, where the outcome of investigation of higher risk nodules and suspected lung cancer is discussed, and treatment planned.
- It is permissible for the SRM and lung cancer MDT to be run simultaneously as a 'hybrid'.

6.2.2 All pulmonary nodules that are indeterminate should be discussed at the LDCT review or Screening Review Meeting. These include:

- nodules that are ≥300mm^3 or ≥8mm diameter with a ≥10% chance of malignancy by Brock score; these usually require a PET-CT for further evaluation
- nodules that show significant growth at interval LDCT.

6.2.3 Note that nodules that only require repeat CT as a further test should be managed by radiologists within the programme, and do not require discussion at MDTs (unless a second opinion is being sought, or prior imaging is available).

6.3 Management by lung cancer service

6.3.1 Referral: LDCT suspicious for lung cancer will receive a screening referral into the suspected lung cancer rapid assessment and diagnosis pathway [8].

6.3.2 Incidental findings: Minor incidental findings are common on LDCT and have the potential to cause increased unnecessary investigations and anxiety to participants. Incidental finding reporting, and management should be based on the following principles:

- The finding should be clinically significant.
- Clinically non-significant findings should not be communicated to the GP or participant.

- Findings that the GP and participant are already aware of, including findings identified on previous LCSP scans, should not be communicated to the GP or participant as actionable findings.
- There should be agreement between the LDCT Lung Cancer Screening Programme and primary care as to the nature and benefit of the recommended interventions.
- Recommendations for clinical correlation by primary care of CT findings should be avoided, and if made, should be specific.

6.3.3 Incidental findings can be broadly categorised as follows:

- Major findings that may be life threatening and should prompt direct referral for admission to hospital by the LDCT Lung Cancer Screening Programme.
- Findings mandating urgent referral (e.g. significantly dilated aortic aneurysm).
- Findings indicative of cancer at another site which should prompt urgent referral via the cancer pathway upgrade process.
- Other non-cancer findings requiring referral to secondary care (e.g. significant fibrotic interstitial lung disease).
- Non-cancer findings that may require management in primary care.
- Other findings that may prompt NICE recommended assessment to be done, where they have not been included in the assessment performed by the RA (e.g. significant coronary calcification on CT may prompt recommendation for cardiovascular Q-Risk assessment).
- Other findings that are usually not directly associated with a beneficial intervention, would not usually be reported by radiologists, or can be deemed clinically non-significant and that do not require communication (e.g. bronchial wall thickening).

6.3.4 Incidental findings will be reviewed by the Screening Review Meeting (SRM) and clear recommendations will be made to the relevant clinicians and to the participant.

6.3.5 The Responsible Radiologist will be accountable for decisions made regarding incidental findings referred to SRM.

6.3.6 There should be a policy agreed between the targeted lung cancer screening site and primary care about management of LDCT findings, including the referral process for incidental findings.

6.3.7 The NHS England LCSP Incidental Findings Management Protocol provides guidance on the management of the most common findings.

7. Non-attendance, moving out of area and exiting the programme

7.1 Non-attendance

7.1.1 First-time attendance should be facilitated by offering LDCT that is easily accessible for the participant (e.g. mobile scanners in community settings, easy transport links, evening and weekend appointments).

7.1.2 The process of changing appointments should be straightforward for those who request this.

7.1.3 There should be a formal process for contacting non-attenders.

7.1.4 Where possible and in line with data protection law, feedback from attenders and non-attenders should be sought to evaluate and improve access.

7.1.5 Participants who do not attend scheduled appointments should be re-invited according to local processes. Participants should be re-invited in the next screening round unless explicitly stating a wish not to be re-invited. If participants have suggested they no longer wish to be invited, the opportunity should remain for them to opt back in to the programme (See 3.1.3).

7.2 Moving out of area

7.2.1 Where possible, sites should identify that a participant has moved address and no longer lives within the LCSP area before any appointment is due, in order to prevent delays.

7.2.2 The site with which a participant sits remains responsible for that participant until the potential receiving site agrees to accept the transfer of that participant to their site, or the participant actively opts-out of the LCSP. If a provider is managing the transfer of a participant, the Cancer Alliance is responsible for ensuring the transfer is completed in a timely manner.

7.2.3 Where a participant moves from one site to another and has already breached the LHC risk threshold, they should not need to complete another Lung Health Check risk assessment.

7.2.4 Every effort should be made to ensure that participants requiring future surveillance (3 month / 12 month) or screening round (24 month) scans receive those scans if they move out of the area covered by the individual LCSP site. This should include, where appropriate, offering the participant the opportunity to return to the original LCSP site for scans.

7.2.5 Where a project is made aware of a participant moving to an area already covered by a Lung Cancer Screening site, the original site should get in touch with the participant's new site to ensure they are added to that programme.

7.2.6 Where a site is made aware of a participant requiring surveillance scans (e.g. for a lung nodule), moving to an area where Lung Cancer Screening is not currently available, they must contact the local hospital Trust to suggest the participant receives surveillance scans in line with this Standard Protocol ahead of Lung Cancer Screening becoming available in the new area. The patient must be informed of the need for further follow up in their new site. Screening round scans should wait until the programme moves to the area and the participant is invited to that programme.

7.2.7 For the avoidance of doubt, any case where a participant has their scan delayed because of moving out of area must be formally raised as an incident with the national LCSP team.

7.2.8 If a participant leaves the country (including moving to another country in the United Kingdom) then they are no longer eligible for the Lung Cancer Screening Programme and should be exited.

7.3 Exiting the programme

7.3.1 All participants should receive at least two LDCT screening round scans (baseline and 24 month scan, but this does not include 3 or 12 month surveillance scans) before ageing out of the programme. For example, if a participant were to receive a prevalent/baseline scan on their 74th birthday where no nodules were found, then they would be invited back for a second screening round scan around their 76th birthday. If that second scan also found no nodules then the participant would exit the programme.

7.3.2 Once a participant turns 75, as long as the participant has received at least two LDCT screening round scans, they should not be booked for any further screening round scan and should exit the programme.

7.3.3 For participants receiving a baseline scan shortly before turning 75, if the scan finds no indeterminate nodules, then the participant will receive one further screening round scan 24 months after their baseline scan. This screening round scan can take place after the participant turns 75.

7.3.4 If indeterminate nodules are found on any screening round scan then surveillance scans (e.g. 3 month or 12 month) should take place, even if these take place after a participant turns 75.

7.3.5 Once a participant over the age of 75 has received two screening round scans and any surveillance scans resulting from those screening round scans, the participant should exit the programme.

7.3.6 Where a participant requires longer follow-up at the final planned surveillance scan e.g. a new nodule or sub-solid nodule, the participant should be referred to the local respiratory service for further follow-up.

7.3.7 If a participant who has not yet received two LDCT screening round scans does not attend a screening round scan and a subsequently booked scan would take place after the participant turns 75, the participant should be given one further opportunity to attend.

7.3.8 If a participant has a CT scan out of programme that necessitates delaying the lung cancer screening LDCT then this can be accommodated, even if that delay extends the LDCTs beyond the point the participant turns 75.

7.3.9 Participants who fail to accept invitations to LHC or CT scans can still attend LHCs or CT scans after turning 75. But no participant should receive a LHC or baseline scan more than 3 months after turning 75 (see 3.1.2).

7.3.10 Under no circumstances should a participant be invited for a screening round scan after turning 78.

7.3.11 The letter informing participants of the outcome of their final LDCT scan should also inform participants that they are being released from the programme.

7.3.12 If a participant leaves the country (including moving to another country in the United Kingdom) then they are no longer eligible for the Lung Cancer Screening Programme and should be exited.

8. Communication of results

8.1 Process

8.1.1 Participants will be sent communication about the results of the LDCT as shown in Appendix A.

8.2 Serious findings

8.2.1 Potentially serious findings will be acted on immediately and more indeterminate findings followed up as required.

8.3 Letters

8.3.1 Standard letters have been prepared, adapted from the UKLS and lung screen uptake randomised controlled trials.

8.3.2 The outcome of the LDCT should be communicated by standard letter to the GP (preferably electronic to facilitate audit) with any action taken or recommended to be taken, included.

8.3.3 SNOMED codes should be used to record the participant journey and outcomes on the participant's GP patient record. National guidelines on SNOMED codes to use for the LCSP should be referred to.

8.3.4 These outcomes should be communicated to the participants by standard letter, except in the unusual circumstance where direct admission is arranged. Letters will not include details of serious findings; this will be explained at clinic visits.

8.3.5 It is expected that template letters provided by the national team should be used in the majority of circumstances to ensure consistency across the programme.

8.4 Telephone

8.4.1 Telephone communication of results by an appropriately trained clinician should be offered as well as communication by letter when clinically appropriate, e.g. serious findings such as malignancy.

8.4.2 There should be a telephone number for participants to phone for further information and clarification when they receive their results.

8.5 Timeframe

8.5.1 The outcome should be communicated within a maximum of four weeks from the LDCT. Safety net processes should be in place to ensure that findings requiring urgent referral are flagged and communicated appropriately.

8.6 General

8.6.1 Generic, non-personalised, information about programmes should be available on the public NHS website.

8.6.2 For participants who are being given a 'normal' result, the possible effect of over-reassurance will be mitigated by including information about continued risk of lung cancer (which may be provided as a percentage based on a multivariable model), the importance of not ignoring red flag symptoms and the importance of not smoking.

8.7 Participant feedback

8.7.1 Following the communication of results to the participant, consideration should be given to offering participants a basic questionnaire about their experience including:

- the lung health check
- booking experience
- consent process
- scanning
- information provided to them
- understanding of the checks process and results process
- willingness to continue in the programme
- the impact of smoking cessation advice on willingness to quit and on participation.

9. Low dose computed tomography data management

9.1 Collection

9.1.1 Data should be collected by the local team in a format that will allow submission to the National Cancer Registration and Analysis.

9.1.2 Where possible and present on CT scanners, a patient dose management system should be used to record data on scans. This should be collected locally in a format that will allow submission to the UK Health Security Agency.

9.2 Handling

9.2.1 All data will be handled in adherence to the Data Protection Act 1998 and information governance legislation. Audit trails will be in place to fully trace data entry and edit.

9.3 Inputting

9.3.1 Inputting of data will comply with information governance legislation.

9.4 Dataset

9.4.1 A minimum mandatory dataset has been agreed. All sites must report data against the minimum dataset to NHS England on a routine basis.

10. Nonadherence with Standard Protocol and governance documents

10.1.1 In some rare instances, it may be necessary to deviate from the Standard Protocol, for example to carry out research. To do this, the Standard Protocol Deviation Form should be completed, any deviation will need to be signed off by the national team SRO and national clinical experts.

10.1.2 If an inadvertent breach of the Standard Protocol is discovered, then this should be considered an incident. The national team should be notified immediately and the LCSP Incident Assessment Form should be completed.

11. Evolution of the standard protocol for the LCSP

11.1 Updating the standard protocol

11.1.1 It is recognised that this targeted screening for lung cancer with low radiation dose computed tomography and standard protocol prepared for the LCSP will evolve over time.

11.1.2 This will be influenced by the LCSP Expert Advisory Group, tasked with providing expert advice, support and guidance to the evaluation of the programme, implementation of the standard protocol, and bringing knowledge and expertise on innovation and developments which would impact on lung cancer outcomes.

11.1.3 Furthermore, this document will need to adapt as further research findings from screening studies emerge.

11.1.4 Advice and consultation with the UKNSC will be ongoing and could influence future iterations of this document.

References

1. Marriott, A. and Turner, S. (2015). Making Reasonable Adjustments to Cancer Screening. [online] London: Public Health England. Available at: https://www.ndti.org.uk/uploads/files/Updated_reasonable_adjustments_in_cancer_scree ning_report.pdf
2. Kovalchik SA, Tammemagi MC, Berg CD, Caporaso NE, Riley TL, Korch M, Silvestri GA, Chaturvedi AK, Katki HA: Targeting of Low-Dose CT Screening According to the Risk of Lung-Cancer Death. New England Journal of Medicine 2013, 369:250
3. Raji OY, Duffy SW, Agbaje OF, Baker SG, Christiani DC, Cassidy A, Field JK: Predictive accuracy of the Liverpool Lung Project risk model for stratifying patients for computed tomography screening for lung cancer: a case-control and cohort validation study. *Annals of Internal Medicine* 2012, 157:242-250.
4. Tammemagi MC, Katki HA, Hocking WG, Church TR, Caporaso N, Kvale PA, Chaturvedi AK, Silvestri GA, Riley TL, Commins J, Berg CD: Selection criteria for lung-cancer screening. *New England Journal of Medicine* 2013, 368:728-736.
5. Ten Haaf K, Jeon J, Tammemagi MC, Han SS, Kong CY, Plevritis SK, Feuer EJ, de Koning HJ, Steyerberg EW, Meza R: Risk prediction models for selection of lung cancer screening candidates: A retrospective validation study. *PLoS Med* 2017, 14: e1002277.
6. Callister, M. Baldwin, D. Akram, A. Barnard, S. Cane, P. Draffan, J. Franks, K. Gleeson,
7. F. Graham, R. Malhotra, P. Prokop, M. Rodger, K. Subesinghe, M. Waller, D. Woolhouse, I. (2015). [online] London: BTS Guidelines for the Investigation and Management of Pulmonary Nodules. Available at: https://www.brit-thoracic.org.uk/document-library/clinical-information/pulmonary-nodules/bts-guidelines- for-pulmonary-nodules/
8. NICE (2011). [online] London: Lung Cancer: diagnosis and management clinical guideline (CG121). Available at: https://www.nice.org.uk/guidance/CG121
9. NHS England (2018). [online] London: Implementing a timed lung cancer diagnostic pathway. Available at: www.england.nhs.uk/wp-content/uploads/2018/04/implementing- timed-lung-cancer-diagnostic-pathway.pdf
10. Wclc2018.iaslc.org. (2018). NELSON Study Shows CT Screening for Nodule Volume Management Reduces Lung Cancer Mortality by 26 Percent in Men. [online] Toronto: The International Association for the Study of Lung Cancer

Appendix A

Participant pathway from invitation, through LDCT, and follow up:

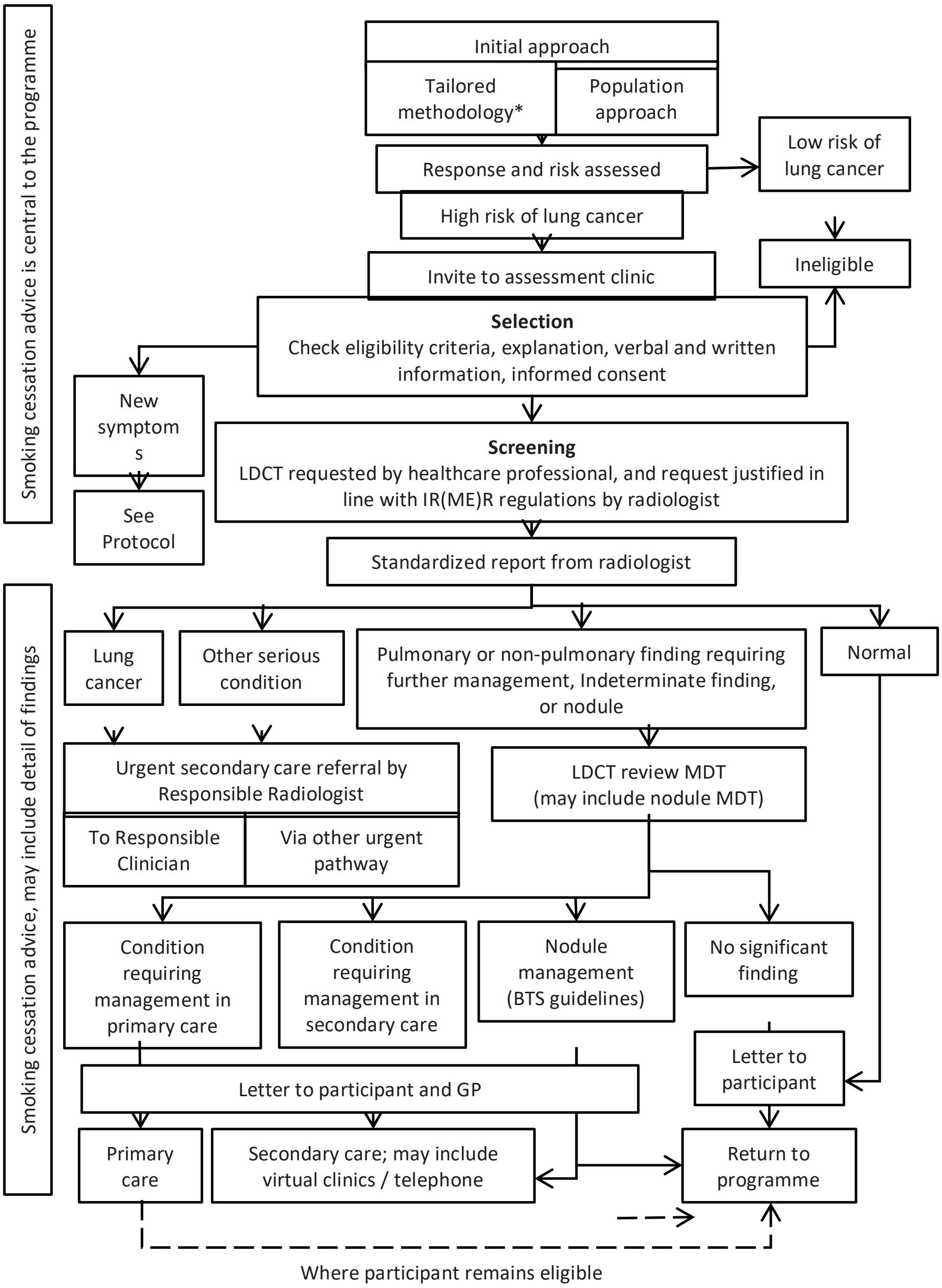

Acknowledgements

The first version of the Standard Protocol was published in January 2019 and developed by the NHS Cancer Programme with the CT Screening Advisory Sub-Group of the Lung Cancer Clinical Expert Group (CEG).

Professor Peter Sasieni	EAG Chair, Deputy Director of the Centre for Cancer Prevention, Queen Mary University, London
Professor David Baldwin	Lung CEG Chair, Consultant in Respiratory Medicine, Nottingham University Hospitals NHS Trust
Dr Sion Barnard	Consultant Thoracic Surgeon, Newcastle upon Tyne NHS Foundation Trust
Dr Richard Booton	Clinical Director for Thoracic Oncology, Manchester University NHS Foundation Trust
Dr Matthew Callister	Consultant in Respiratory Medicine, The Leeds Teaching Hospitals NHS Trust
Dr Phil Crosbie	Consultant in Respiratory Medicine, Manchester University NHS Foundation Trust
Dr Anand Devaraj	Thoracic Radiologist, Royal Brompton Hospital & Harefield NHS Foundation Trust
Tim Elliott	Senior Policy Advisor, Department of Health
Siobhan Farmer	Public Health Consultant, Screening and Immunisation Lead, Greater Manchester Health and Social Care Partnership
Professor John Field	Professor of Molecular Oncology, University of Liverpool
Dr Jesme Fox	Medical Director, Roy Castle Lung Cancer Foundation
Martin Grange	Patient Representative

Dr John Holemans	Consultant Radiologist, Liverpool Heart and Chest Hospital NHS Foundation Trust
Professor Sam Janes	Vice Chair Lung CEG, Consultant in Respiratory Medicine, University College London Hospitals NHS Foundation Trust
Dr Richard Lee	Consultant Respiratory Physician, Royal Marsden NHS Foundation Trust
Dr Jodie Moffat	Head of Early Diagnosis, Cancer Research UK
Dr Arjun Nair	
Professor Mick Peake	Clinical Director, Centre for Cancer Outcomes, University College London Hospitals Cancer Collaborative
Dr Amelia Randle	GP, Clinical Lead, Somerset, Wiltshire, Avon and Gloucestershire Cancer Alliance
Janette Rawlinson	Patient Representative
Dr Robert Rintoul	Respiratory Physician, Royal Papworth Hospital NHS Foundation Trust
Dr Anna Sharman	Thoracic Radiologist, Manchester University NHS Foundation Trust
Matthew Legg	Programme Manager for Early Diagnosis, NHS England
Charis Stacey	Senior Programme Manager for Early Diagnosis, NHS England

2024 Edits to the Standard Protocol have been overseen by the LCSP Expert Advisory Group and include contributions from:

A Marks
Amelia Randle
Anand Devaraj
Anne Mackie
Anne Stevenson
Arjun Nair
Charlotte Graham
Ciaran Osborne

David Baldwin
Dilek Demirhan
Janette Rawlinson
Jesme Fox
Jodie Moffat
Katherine Brain
Liz Rochelle
Martin Grange
Matthew Callister
Michelle Clark
Neil Navani
Natali Garcia Gillam
Nicola McMaster
Peter Johnson
Peter Sasieni
Phillip Crosbie
Poppy Richards
Rebecca Towers
Richard Lee
Sam Janes
Samantha Quaife
Tim Windle

NHS England
Wellington House
133-155 Waterloo Road
London
SE1 8UG

Contact: england.TLHC@nhs.net

This publication can be made available in a number of alternative formats on request.

© NHS England 2022 | PR1646

Classification: Official

Publication reference: PR1647

NHS England

Targeted screening for lung cancer with low radiation dose computed tomography

Quality assurance standards prepared for the Lung Cancer Screening Programme

Version 3, 3 February 2025

Prepared with guidance from the Lung Clinical Expert Advisory Group

Contents

Introduction 2

Standard 1: Lung cancer screening – nursing and support staff 3

Standard 2: Lung cancer screening – radiologists..5

Standard 3: Radiology hardware 7

Standard 4: Radiology software...8

Standard 5: Patient administration system software..10

Standard 6: Data management..11

Standard 7: Lung health checks programme pathway 13

Standard 8: Participant communications 15

Standard 9: General practice communications..17

Standard 10: Smoking cessation 18

Standard 12: Low dose CT referral...19

Standard 13: Low dose CT reporting..21

Standard 14: Quality assurance of low dose CT scans 22

Standard 15: External quality assurance of radiologists...25

Annex 1: Low dose CT reporting proforma...27

Annex 2: Protocol for the management of incidental findings in lung cancer screening 34

Table 1: Incidental findings, reporting and management, based on latest evidence*. 39

Annex 3: National incidental findings pathways for targeted lung cancer screening 49

Introduction

1.1. The national Lung Cancer Screening Programme offers people aged 55 to 74 an opportunity to participate. Those who have ever smoked will be eligible to have a lung health check; and for those at risk of lung cancer, a referral to lung cancer screening with a low-dose computed tomography (LDCT) scan of the chest. The programme contributes to the overall Long Term Plan early diagnosis of cancer ambition, stating that by 2028 the proportion of cancers diagnosed at stage one and two will rise to three quarters of cancer patients.

1.2. This document sets out 15 quality standards for the programme that together form the quality assurance framework for skills and training, information and communication, and clinical delivery. The quality standards assurance framework sets the standards for staffing, nurse and radiologist qualifications, experience and training, hardware, software, data management, communications, radiology acquisition and reporting, and follow on clinical management in secondary care.

1.3. Each standard relates to a specific part of the targeted lung health check pathway and cross references to the published standard protocol. Each standard sets out the objective, definition and metric, and the local and national assurance and audit process to demonstrate that each standard is being met.

1.4. The standard protocol outlines the four clinical roles each project must have in place to ensure the effective delivery of care and clinical governance of the programme. The clinical director of programme will work with the responsible assessor, responsible radiologist and responsible clinician to implement and monitor the 15 quality standards.

1.5. Each project will establish local processes to ensure the quality standards are continually met. The clinical director of programme will report against these standards on a quarterly basis to NHS England. An annual summary report should be drawn from this quarterly data, incorporating additional metrics better suited to annual review.

Standard 1: Lung cancer screening – nursing and support staff

Cross reference to Lung Cancer Screening Programme Standard Protocol – section 2.3.4.

1a. Description

This standard sets out the training and experience requirements for nurses and support staff who conduct lung health checks and manage the lung cancer screening programme.

1b. Objective

- To ensure that the project has the trained and skilled workforce with the capacity to deliver the programme.
- To ensure nurses and support staff delivering the Lung Cancer Screening Programme are qualified and competent.
- To ensure the service is safe and effective.

1c. Definition

Minimum qualifications for nurses:

- NHS Band 6 qualified.
- Registered with the Nursing and Midwifery Council.
- For those performing spirometry to Association for Respiratory Technology and Physiology (ARTP) guidelines, on the national spirometry register (relevant for all healthcare practitioners performing spirometry).

Minimum training course requirements for nurses:

- Communicating with high-risk individuals about lung cancer screening (offered nationally).
- Consent training (not offered nationally).
- Ionising radiation (medical exposure) regulations [IR(ME)R] for referrers (not offered nationally).
- Locally designed training covering telephone assessment process, call quality expectations and control measures, including identification of red flag symptoms.

Minimum qualifications for support staff:

- NHS Band 3 qualified.

Minimum training course requirements for support staff:

- Communicating with high-risk individuals about lung cancer screening.
- Very Brief Advice (smoking cessation) training
- Locally designed training covering telephone assessment process, call quality expectations and control measures, including identification of red flag symptoms.

1d. Metric

- 100% of nurses and support staff conducting lung health checks meet the minimum qualifications and minimum training course requirements.
- 100% of those conducting spirometry are on the national spirometry register.
- A record is maintained to show the % of lung health checks that are re-categorised from low to high risk or vice versa following local audit.

1e. Local audit

The clinical director of the programme will ensure nurses and support staff providing direct care meet the minimum training standard and for practitioners performing spirometry. They will maintain a local minimum training and experience record for nurses and other healthcare practitioners. The quality assurance process should include an audit of the accuracy of 50 or 1% (whichever is smaller) of telephone screening assessments conducted per quarter.

1f. National audit

The clinical director of the programme will report quarterly against this standard to the Lung Cancer Screening Programme Delivery Group and through the quarterly quality assurance process.

Training courses

Training courses are available to demonstrate competence to perform lung health checks, spirometry and to meet the IR(ME)R regulations for referral to computerised tomography (CT).

Standard 2: Lung cancer screening – radiologists

Cross reference to Lung Cancer Screening Programme Standard Protocol – section 4.6.1.

2a. Description

This standard sets out the training and experience requirements for radiologists who report low dose CT lung cancer screening scans for the Lung Cancer Screening Programme.

2b. Objective

- To ensure that the project has the trained and skilled workforce with the capacity to deliver the programme.
- To ensure consultant radiologists reporting low dose CT lung cancer screening are qualified and competent.
- To ensure the service is safe and effective.

2c. Definition

Minimum qualifications for consultant radiologists:

- Registered with the General Medical Council (GMC).
- Fellow of the Royal College of Radiologists (RCR).
  - In the absence of the above qualifications, consultant radiologists who:
    - are on the General Medical Council (GMC) Specialist Register; or
    - have radiology training and qualification accepted for equivalence which has led to the award of a Certificate of Eligibility for Specialist Registration (CESR)
    - can report for the programme subject to approval by the clinical director and responsible radiologist of the project

Minimum training course requirements:

- Lung Nodule Identification Workshop (run by NHS England).

Minimum experience:

- Reporting a minimum of 500 thoracic CTs per annum in their routine clinical practice
  - a significant proportion of the CTs are where there is a suspicion of lung cancer.

- Regular participation at a thoracic multidisciplinary training (MDT) meeting (includes virtual attendance) as part of their routine clinical work.

The responsible radiologist must be satisfied that evidence of all the above has been provided before a radiologist is permitted to report for the programme.

2d. Metric

- 100% of consultant radiologists reporting thoracic low dose CT scans for the Lung Cancer Screening Programme meet the minimum requirements.

2e. Local audit

The responsible radiologist will ensure reporting radiologists always meets the minimum standard. They will maintain a local minimum training and experience record for radiologists reporting low dose CT scans for the programme.

2f. National audit

The clinical director of the programme will report quarterly against this standard to the Lung Cancer Screening Programme Delivery Group and through the quarterly quality assurance process.

Training course: Lung nodule workshop

The British Society of Thoracic Imaging (BSTI) provides training events for radiologists to gain specific competency and experience in reading low dose CT lung cancer screening scans.

Standard 3: Radiology hardware

Cross reference to Lung Cancer Screening Programme Standard Protocol – sections 4.1.1 and 4.3.1.

3a. Description

This standard sets out the hardware requirements for CT scanners used to deliver the Lung Cancer Screening Programme.

3b. Objective

- To ensure CT scanning equipment is safe and effective.
- To ensure harm from radiation is minimised by using as low a dose of radiation as possible.
- To ensure image quality will allow radiologists to detect lung cancers.

3c. Definition

Minimum standard:

- A sixteen channel multi-detector CT, fixed site or mobile, and calibrated according to the manufacturer's specifications, capable of delivering low radiation dose protocols.
- The calculated radiation dose delivered to each individual is below 2 mSv (based on a median standard 70kg adult).

3d. Metric

- Medical physics expert's (MPE) confirmation that the scanner meets the minimum standard.
- 100% of radiation doses meet the minimum standard.

3e. Local audit

The local MPE will perform regular radiation dose audit. The responsible radiologist will work with the local MPE to ensure the low dose CT scanner always meets the minimum standard.

3f. National audit

The clinical director of programme will report quarterly against this standard to the Lung Cancer Screening Programme Delivery Group and through the quarterly quality assurance process.

Standard 4: Radiology software

Cross reference to Lung Cancer Screening Programme Standard Protocol – sections 4.1 and 4.4.

4a. Description

This standard sets out the software requirements for reporting low dose CT scans.

4b. Objective

- To ensure the reporting radiology environment and process is efficient, using software that assists in producing rapid and accurate reports.
- To ensure auto-population of participant demographic data, scan parameter data, Brock scores and dates of scans into reporting proforma to prevent human error and reduce reporting time.

4c. Definition

Analysis and reporting software, including voice recognition reporting software, is compatible with data acquisition requirements. Volumetric software used for assessment of pulmonary nodules remains constant to allow accurate comparison of volumes.

If software upgrades or changes are made the new software will remeasure the old and follow up nodules unless data is available to demonstrate consistency between models.

Minimum standard:

- Computer-aided detection.
- Nodule volumetry software that automatically detects nodules and measures volume.
- Ability to retrieve and compare any previous CT imaging.

Desirable standard:

- Facilitates double reads.

4d. Metric

- 100% of image reconstruction is standardised and used for any subsequent follow-up examinations where possible with emphasis on ensuring that slice thickness, reconstruction increment, reconstruction algorithm is identical.

- 100% of slice thickness are ≤ 1.25mm.[1]

4e. Local audit

The responsible radiologist will ensure the reporting software always meets the minimum standard.

4f. National audit

The clinical director of programme will report quarterly against this standard to the Lung Cancer Screening Programme Delivery Group and through the quarterly quality assurance process.

[1] Examples of reconstruction parameters used in low-dose screening CT for moderate spatial frequency/soft tissue are: reconstruction slice thickness 1mm; reconstruction increment 0.7mm; reconstruction FOV of the entire lung parenchyma.

Standard 5: Patient administration system software

Cross reference to Lung Cancer Screening Programme Standard Protocol – section 3.

5a. Description

This standard sets out the software requirements for the patient administration system that projects will use to call and re-call participants invited to the Lung Cancer Screening Programme.

5b. Objective

- To ensure participants invited and all subsequent appointments are managed through an auditable patient administration system.
- To prevent harm to participants caused by failure to recall or to follow up on findings.

5c. Definition

Patient administration software will support participant administration that is reliable and delivers a consistent process which facilitates recall, governance, audit and evaluation. Software should align with the minimum requirements set out in any specification provided by the national team.

5d. Metric

- Patient administration system and software meets the minimum standard.

5e. Local audit

The responsible assessor will ensure the patient administration systems used to deliver the lung health checks programme meet the minimum standard.

5f. National audit

The clinical director of the programme will report quarterly against this standard to the Lung Cancer Screening Programme Delivery Group and through the quarterly quality assurance process.

Standard 6: Data management

Cross reference to Lung Cancer Screening Programme Standard Protocol – section 9.

6a. Description

Standard sets out what data sharing agreements and pseudonymisation processes are in place to control and manage participant data.

6b. Objective

- To ensure data processing agreements and data sharing agreements are in place to direct how participant data is recorded, handled and used to deliver the Lung Cancer Screening Programme.
- To ensure the confidentiality of participant data.
- To ensure relevant data is pseudonymised.
- To ensure that processes are accessible to future research requests.

6c. Definition

Projects will ensure local Data Protection Impact Assessments (DPIAs), Data Processing Agreements (DPAs) and Data Sharing Agreements (DSAs) are agreed, detailing how data is collected and used to deliver the project, and shared with Data Services for Commissioners Regional Offices (DSCRO).

The projects will work with the DSCRO to establish a process to pseudonymise the minimum dataset. DPIA and DSA will be considerate of the need for future accessibility of data that may be required for research purposes.

6d. Metric

- Any necessary Data Processing Agreements agreed.
- Data Sharing Agreements agreed.
- 100% adherence to local and national DPIA processes, including pseudonymisation.

6e. Local audit

The clinical director of the programme will ensure that data management always meets the minimum standard.

6f. National audit

The clinical director of the programme will report quarterly against this standard to the Lung Cancer Screening Programme Delivery Group and through the quarterly quality assurance process.

Standard 7: Lung health checks programme pathway

Cross reference to Lung Cancer Screening Programme Standard Protocol – sections 3 to 8.

7a. Description

This standard sets out what will happen in the lung health checks pathway from the identification of eligible participants, the lung health check, lung cancer risk assessment, smoking cessation and low dose CT scanning through to follow up.

7b. Objective

- To ensure the clinical teams adhere to and ensure accuracy across the lung health checks programme pathway.
- To ensure all participants receive the same level of interventions and care, and opportunities for face to face conversations about lifestyle changes and especially smoking cessation, are maximised.

7c. Definition

The lung health checks programme pathway is shown in figure 1 over the page:

Figure 1: Lung health checks programme pathway

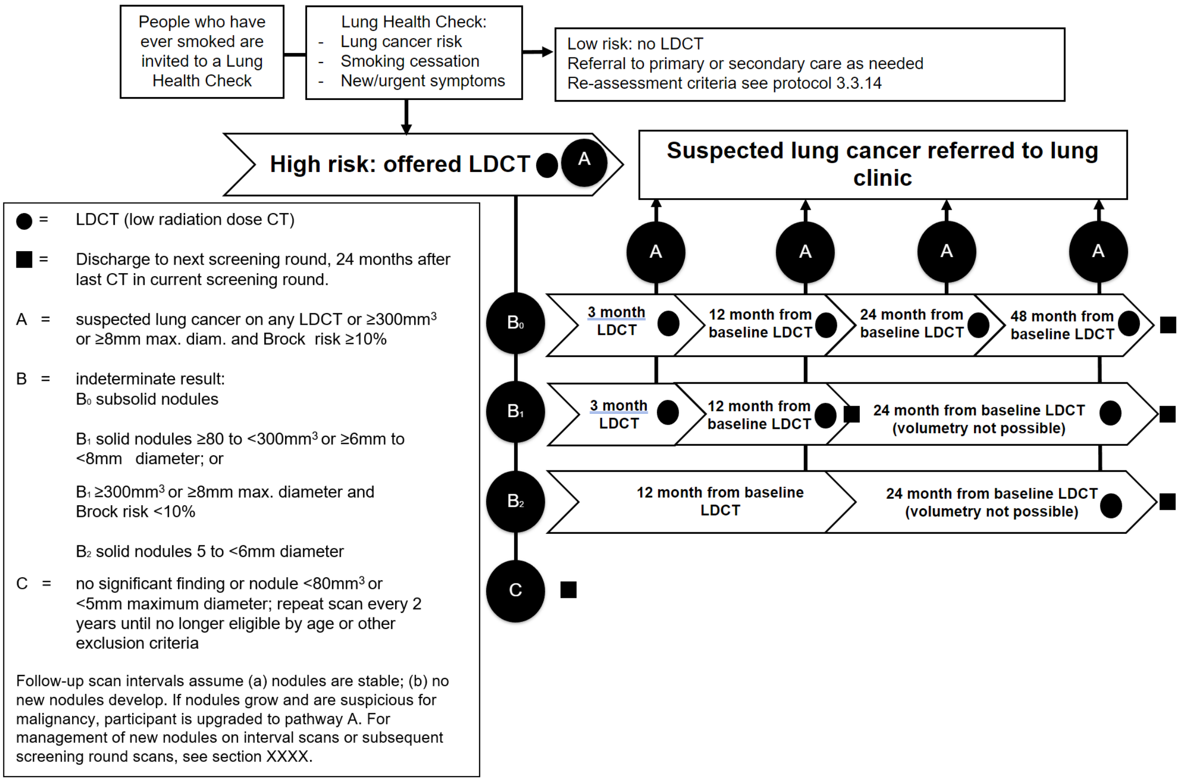

7d. Metric

- 100% of participants follow the lung health checks programme pathway.

7e. Local audit

The responsible assessor will ensure participants follow the lung health checks programme pathway and that the lung health check always meets the minimum standard.

7f. National audit

The clinical director of programme will report quarterly against this standard to the Lung Cancer Screening Programme Delivery Group and through the quarterly quality assurance process.

Standard 8: Participant communications

Cross reference to Lung Cancer Screening Programme Standard Protocol – sections 3.1, 3.4 and 8.

8a. Description

This standard sets out what information participants will receive: from the point of invitation, results and onward referral, up to the point of discharge.

8b. Objective

- To ensure that the site accurately identifies the population eligible for targeted screening.
- To ensure participants are provided with information to allow them to make an informed decision to maximise uptake in the eligible population.
- To ensure communication relating to invitation approach, results, referrals and discharge is consistent across the programme to maximise informed choice at each step of the pathway.

8c. Definition

The issuing of the standard letters[2] and the participant booklet is detailed in figure 2 over the page:

[2] The standard letters and participant booklet are available on request from england.TLHC@nhs.net.

Figure 2: Issuing of standard letters and participant booklet

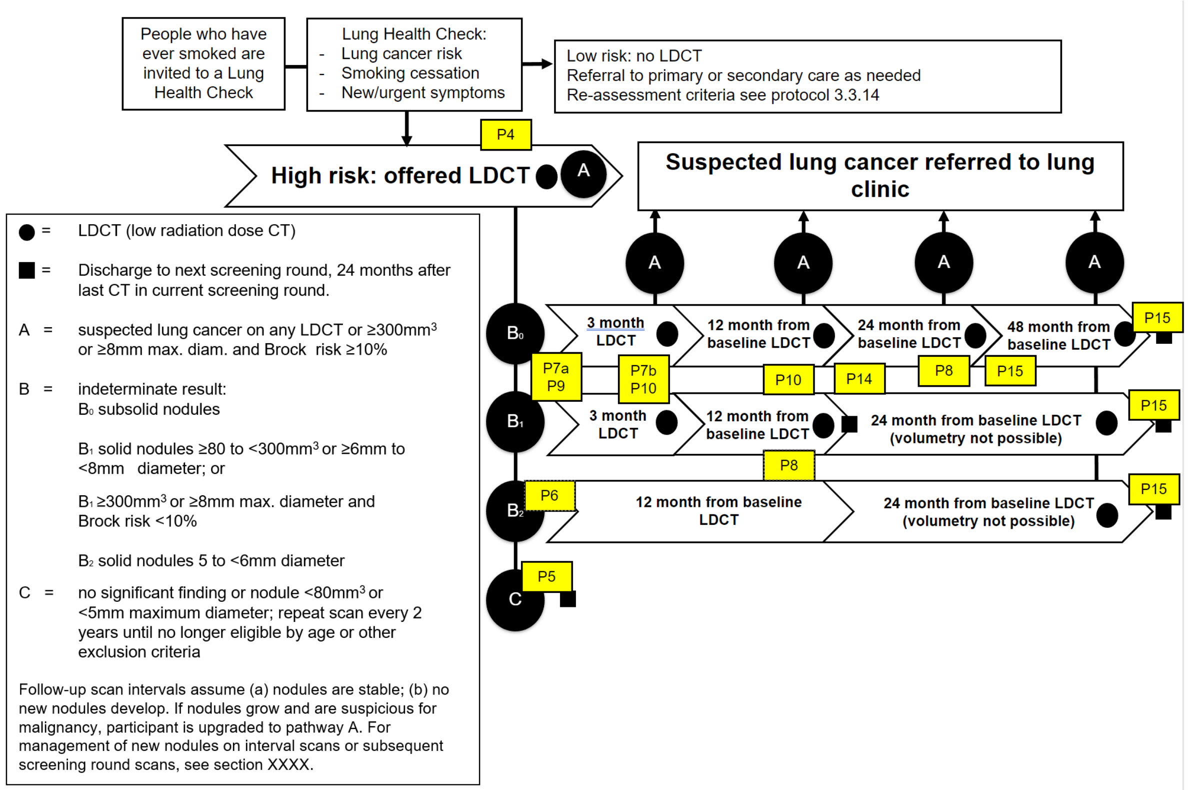

8d. Metric

- 100% of participants will receive the standard letters and the standard booklet at the correct point in the pathway.
- 100% of participants who attend the lung health check or have a CT scan will receive an outcome letter within 28 days of an appointment
- 100% of participants who receive a technically adequate LDCT will receive an outcome letter within 28 days of LDCT acquisition.

8e. Local audit

The responsible assessor will ensure that communication methods always meet the standard.

8f. National audit

The clinical director of the programme will report quarterly against this standard to the Lung Cancer Screening Programme Delivery Group and through the quarterly quality assurance process.

Standard 9: General practice communications

Cross reference to Lung Cancer Screening Programme Standard Protocol – sections 3.1, 3.4 and 8.

9a. Description

This standard sets out what information a participant's GP will receive.

9b. Objective

- To ensure that GPs have all the information on whether a participant attended a lung health check, the outcome of this and subsequent follow up.
- To ensure the effective management of significant incidental findings that are agreed locally and set out in project clinical pathways.

9c. Definition

Letters to a participant's GP must include details of new results from the lung health check appointment (lung health check assessment, risk assessment, and smoking cessation or any other lifestyle advice), relevant low dose CT scan information and the plan of care. The issuing of the standard letters[3] to GPs is detailed in figure 2 above.

9d. Metric

- 100% of GP letters includes the minimum standard information.
- 100% of GP letters are sent within 28 days of the participant attending an appointment or scan.

9e. Local audit

The responsible assessor will ensure that the minimum standard is always met.

9f. National audit

The clinical director of the programme will report quarterly against this standard to the Lung Cancer Screening Programme Delivery Group and through the quarterly quality assurance process.

[3] The standard template is available on request from england.cancerpolicy@nhs.net.

Standard 10: Smoking cessation

Cross reference to Lung Cancer Screening Programme Standard Protocol – sections 3.2.2 and 3.4.

10a. Description

This standard sets out the expectations for offering smoking cessation interventions as part of the Lung Cancer Screening Programme.

10b. Objective

- To ensure the opportunities for educating, counselling and supporting participants to quit smoking are maximised.
- To ensure lung health check nurses offer opt-out referral to local smoking cessation services to participants that are current smokers.
  - Smoking cessation support should be offered to all participants at their lung health check, including those who are ineligible for LDCT.
  - Where possible this should be provided in the immediate lung health check setting and include offer of pharmacotherapy.

10c. Definition

The uptake of smoking cessation courses and quit rates.

10d. Metric

- 100% of current smokers that attend a lung health check are offered a smoking cessation intervention.

10e. Local audit

The responsible assessor will ensure that smoking cessation interventions are offered to all current smokers who attend a lung health check.

10f. National audit

The clinical director of programme will report quarterly against this standard to the Lung Cancer Screening Programme Delivery Group and through the quarterly quality assurance process.

Standard 12: Low dose CT referral

Cross reference to Lung Cancer Screening Programme Standard Protocol – section 3.3.

12a. Description

This standard sets out how participants with a positive lung cancer risk score are identified and referred for a low dose CT scan.

12b. Objective

- To ensure only participants that are at risk of lung cancer are referred for a low dose CT scan.
- To ensure that the CT scan is acquired at the earliest opportunity following the lung health check appointment.
- To ensure follow up CT scans are acquired as detailed in the participant's clinical record.

12c. Definition

A participant will proceed to lung cancer screening if they meet the minimum threshold of either the Liverpool Lung Project or the Prostate Lung Colorectal and Ovarian risk prediction tool. Each tool assesses risk as follows:

- Liverpool Lung Project (LLPv2) ≥2.5% risk of lung cancer over five years

or:

- Prostate Lung Colorectal and Ovarian or ($PLCO_{m2012}$) ≥1.51% risk of lung cancer over six years.

A participant who scores positive using either risk prediction model and does not meet any of the exclusion criteria will receive a low dose CT scan within 56 days of their lung health check.

Participants who require a follow up surveillance low dose CT scan will receive this within 28 days after the target date for the scan.

Participants invited for a subsequent screening round scan should receive this within 56 days before or after their planned date for the routine screening round.

12d. Metrics

- 100% of those referred for a low dose CT scan have a risk prediction score of LLPv2 ≥2.5% over five years or $PLCO_{m2012}$ ≥1.51% risk of lung cancer over six years.
- Percentage of participants who have the CT scan on the same day as their lung health check.
- For those who do not have same day CT, the length of time from lung health check to CT scan in days.
- Audit follow up surveillance scans that are not are completed within the 28-day window after target surveillance follow up scan date.

Audit incident screening round scans that are not completed withing 56 days of the target screening round scan date.

12e. Local audit

The responsible radiologist will ensure that the referral for lung cancer screening always meets the minimum standard. The responsible assessor will audit all participants that have a surveillance follow-up scan outside the 28-day window, or incident screening round scans beyond 56 days from the target date, and agree an action plan to reduce the number of scans acquired off plan.

12f. National audit

The clinical director of the programme will report quarterly against this standard to the Lung Cancer Screening Programme Delivery Group and through the quarterly quality assurance process.

Standard 13: Low dose CT reporting

Cross reference to Lung Cancer Screening Programme Standard Protocol – section 4.6.

13a. Description

This standard sets out how low dose CT scans are reported.

13b. Objective

- To ensure reporting of low dose CT scans are consistent and standardised.
- To ensure radiologists clinically report, using the incidental findings guidance for each participant.

13c. Definition

Radiologists will use the low dose CT reporting proforma in Annex 1. Radiologists will report incidental findings using the guidance in Annex 2.

The overall target for referral is <15%. The referral rate is a combination of referrals for suspected lung cancer via fast track clinic, including nodules requiring work-up other than additional LDCT (eg PET-CT), target <7% [Annex 1, nodules 1-3]; and referral for significant incidental findings (<8%) [Annex 1, nodules 1, 4]. Significant incidental findings are defined in Annex 2 along with non-significant incidental findings.

13d. Metric

- 100% of CT reports for the Targeted Lung Health Check programme contain the information detailed in the CT reporting proforma.
- 100% of radiologists use the incidental finding management protocol to inform interpretation of low dose CT scans.
- Overall project referral rates are <15%.

13e. Local audit

The responsible radiologist will ensure that reporting proforma and management of incidental findings process is followed, and that the overall referral rates are <15%.

13f. National audit

The clinical director of programme will report quarterly against this standard to the Lung Cancer Screening Programme Delivery Group and through the quarterly quality assurance process.

Standard 14: Quality assurance of low dose CT scans

Cross reference to Lung Cancer Screening Programme Standard Protocol – sections 4.3 and 4.6.2.

14a. Description

This standard sets out the quality assurance of the acquisition and reporting of low dose CT scans.

14b. Objective

- To ensure participants receive low dose CT scans of diagnostic quality with no excessive radiation.
- To ensure radiologists are supported by peers to improve the quality of reporting low dose CT scans.

14c. Definition

- Acquisition of low dose CT scans:
  - Standard 3 defines the acquisition requirements that radiographers must adhere to.

- Double reporting:
  - the first 25 CT scans reported by each radiologist in a lung health check programme are double read. Double reading is performed by radiologists within the same lung health check programme**.** Where there are discrepancies between reporting decisions, the responsible radiologist should discuss with the clinical director of programme to agree the mechanism for arbitration.

- Quarterly and annual reviews:
  - the responsible radiologist will review reporting performance on a quarterly and annual basis. They will work with the clinical director of programme to support radiologists who are outliers.

14d. Metric

100% of scans are of diagnostic quality

- Audit and review the non-diagnostic CT quality rate.
- Audit and review reasons for all radiation doses greater than 2 mSv.

100% of reporting radiologists have quarterly and annual reviews.

Quarterly review

Audit the mean, standard deviation, median, interquartile and range of the following metrics for each radiologist:

- numbers reported
- recall rates to secondary care for nodules
- recall rates to secondary care for incidental findings
- number of referrals considered inappropriate by the screening or lung cancer MDT (for direct feedback)
- number of additional investigations generated for incidental findings per participant
- number of PET-CTs performed
- benign biopsies
- benign resections
- interval cancer rates
- sensitivity
- specificity.

Annual review

In addition to the quarterly metrics, includes a review of:

- training and experience standards (Standard 2)
- the number of screening scans reported per programmed activity
- incidental finding rate, divided into non-significant incidental findings and significant incidental findings
- lung nodule rate, the number and percentage of:
  - nodules referred for investigation in secondary care
  - indeterminate nodules requiring additional LDCT surveillance at a rate of 11-20% [Annex 1, nodules 1-3]
  - nodules requiring no action (false positives).

100% of outliers, as defined from a quarterly or annual review, will have evidence of agreed actions (including a period of double reporting) with the responsible radiologists.

14e. Local audit

The responsible radiologist will ensure that the quality assurance of the acquisition and reporting low dose CT is followed, and quarterly and annual reviews are completed. The responsible radiologist and responsible clinician will compile an annual report on the mean, standard deviation, median, interquartile and range of the aggregate quarterly metrics.

14f. National audit

The clinical director of programme will report quarterly against this standard to the Lung Cancer Screening Programme Delivery Group and submit an annual quality assurance report on the acquisition and reporting of low dose CT scans.

Standard 15: External quality assurance of radiologists

15a. Description

Reporting radiologists will undertake an annual external quality assurance programme (PERFECTS) to read low dose CT scans. This will involve radiologists completing 100 exercises with the results used to benchmark reporting of radiologists with peers. The programme will establish a feedback loop to measure the ongoing quality of radiologists reporting practices.

15a. Objective

To ensure reporting of low dose CT scans is evaluated to flag outliers who have high rates of recalls and high rates of interval cancers being detected. To ensure radiologists that are outliers receive training and ongoing support overseen by the responsible radiologist and clinical director of the programme.

15b. Definition

Any radiologist reporting on the Targeted Lung Health Check programme must complete the PERFECTS assessment at least once a year to be able to continue reporting on the programme.

The assessment involves tasks focused on; detection and interpretation; focused interpretation and baseline nodule management; and nodule management at follow up. It takes between 4 and 6 hours to complete and can be completed in stages.

15c. Metrics

- 100% of radiologists reporting complete the PERFECTS assessment at least once per year (newly reporting radiologists starting after the annual deadline should complete PERFECTS within 3 months of starting)

15d. Local audit

The responsible radiologist is responsible for ensuring that all radiologists reporting on the programme have completed PERFECTS training. The responsible radiologist will receive

notification of any radiologists that are outliers and who may need additional support or training.

15e. National audit

The clinical director of the programme will report quarterly against this standard to the Lung Cancer Screening Delivery Group and through the quarterly quality assurance process.

Annex 1: Low dose CT reporting proforma

This reporting template captures all findings in a structured format and provides an example of how this may look. Radiology departments will use this annex to create a structured automated report template in the radiology reporting system currently or hosted as an electronic form.

Commercially available lung cancer screening reporting software will report nodule and other findings in a PDF format and a digital imaging and communications in medicine (DICOM) capture object.

Radiologists will need to report incidental findings not included in the reports from the commercial software once transferred to the picture archiving and communications system (PACS) or exported in an extended markup language (XML) format.

In setting up the programme, the responsible radiologist, the clinical director of programme, local PACS and information technology teams will agree which format is used to capture, store and communicate the report.

Field description	Variable input options	Type of input[4]
Radiologist	Name	Autopopulated
GMC Number	GMC Number	Autopopulated
Site of LDCT	Autopopulated from DICOM descriptor (StationName, DICOM tag 0008,1010) for the individual CT scanner	Autopopulated
Type of scan	Baseline/ 3 month/ 12 month/ 24 month	Dropdown
Date of Scan	Autopopulated from DICOM descriptor (StudyDate, DICOM tag 0008, 0020)	Autopopulated
Date of Report	Autopopulated from Reporting Solution	Autopopulated
**Was computer-aided detection (CAD) available?**	Yes/ No - software failed to process study/ No - other (specify)	Dropdown
**Scan quality**	Adequate/Inadequate due to breathing artefact/Inadequate coverage	Dropdown
Participant Name	Autopopulated from DICOM descriptor (PatientName, DICOM tag 0010,0010)	Autopopulated
Participant unique ID	Autopopulated from DICOM descriptor (PatientID, DICOM tag 0010,0020)- should be NHS number	Autopopulated
Age	Autopopulated from XML from nodule reading software or calculated from DICOM (date of current scan- date of birth)	Autopopulated
Sex	Autopopulated	Autopopulated
History of Extra-Thoracic cancer	No/Yes	Dropdown
Family history of lung cancer[5]	No/Yes	Dropdown
**Nodule1**		
Nodule1_sliceNo	Slice from series used for volumetry	Free text
Nodule1_Volumetry reliable?	Yes/No	Dropdown
Nodule1_Nodule size (mm3)	Nodule volume	Free text

[4] Type of inputs: “dropdown” denotes a field where variables could be inputted as a dropdown menu for the reporting radiologist to choose the correct option, where the reporting tool allows for such a function.
[5] Include ‘History of extrathoracic cancer’ and ‘Family history of cancer’ into the referral for low dose CT, as this information is required by the reporting radiologist. This could be done by, for example, ensuring this information is visible in the electronic or paper request form used to request the CT, or providing access to the lung health check questionnaire answers provided by the participant.

Field description	Variable input options	Type of input[4]
Nodule1_maximum diameter (mm)	Nodule longest diameter	Free text
Nodule1_Nodule type	pure ground-glass/part-solid/solid/ IPLN/inflammatory consolidation	Dropdown
Nodule1_Lobe	RUL/RML/RLL/LUL/LLL	Dropdown
Nodule1_Position	intraparenchymal/subpleural/endobronchial	Dropdown
Nodule1_Spiculated	No/Yes	Dropdown
Nodule1_suspicious features	none/bubble-like appearance/ air bronchogram/ pleural indentation/ pleural retraction/ cyst with irregular wall	Dropdown
(multiple selections possible)		
Nodule1_Brock score[6]	Brock score	Autopopulated
Nodule1_change assessment	Growth (Volume change from baseline >25% if volume reliable=Yes, OR diameter change>2mm if volume reliable=No)/ stable/ shrinking/ resolved/ NEW	Dropdown
Nodule1_VDT (days)	Volume doubling time from baseline	Free text
**Use same reporting fields for Nodule 2, 3 and 4 (if applicable)**		
Nodule2_sliceNo	Slice from series used for volumetry	Free text
Nodule2_Volumetry reliable?	Yes/No	Dropdown
Nodule2_Nodule size (mm3)	Nodule volume	Free text
Nodule2_Nodule maximum diameter (mm)	Nodule longest diameter	Free text
Nodule2_Nodule type	pure ground-glass/part-solid/solid/ IPLN/inflammatory	Dropdown
Nodule2_Lobe	RUL/RML/RLL/LUL/LLL	Dropdown
Nodule2_Position	intraparenchymal/subpleural/endobronchial	Dropdown
Nodule2_Spiculated	No/Yes	Dropdown
Nodule2_ suspicious features	none/bubble-like appearance/ air bronchogram/ pleural indentation/ pleural retraction/ cyst with irregular wall	Dropdown
(multiple selections possible)		
Nodule2_Brock score[6]	Brock score	Autopopulated

[6] Brock score is calculated automatically in commercial lung cancer screening reporting software.

Field description	Variable input options	Type of input[4]
Nodule2_change assessment	Growth (Volume change from baseline >25% if volume reliable=Yes, OR diameter change>2mm if volume reliable=No)/stable/ shrinking/ resolved/NEW	Dropdown
Nodule2_VDT (days)	Volume doubling time from baseline	Free text
**Nodule3**		
Nodule3_sliceNo	Slice from series used for volumetry	Free text
Nodule3_Volumetry reliable?	Yes/No	Dropdown
Nodule3_Nodule size (mm3)	Nodule volume	Free text
Nodule3_Nodule maximum diameter (mm)	Nodule longest diameter	Free text
Nodule3_Nodule type	pure ground-glass/ part-solid/ solid/ IPLN/inflammatory	Dropdown
Nodule3_Lobe	RUL/RML/RLL/LUL/LLL	Dropdown
Nodule3_Position	intraparenchymal/subpleural/endobronchial	Dropdown
Nodule3_Spiculated	No/Yes	Dropdown
Nodule3_ suspicious features	none/bubble-like appearance/ air bronchogram/ pleural indentation/ pleural retraction/ cyst with irregular wall	Dropdown
(multiple selections possible)		
Nodule3_Brock score[6]	Brock score	Autopopulated
Nodule3_change assessment	Growth (Volume change from baseline >25% if volume reliable=Yes, OR diameter change>2mm if volume reliable=No)/stable/ shrinking/ resolved/NEW	Dropdown
Nodule3_VDT (days)	Volume doubling time from baseline	Free text
**Nodule4**		
Nodule4_sliceNo	Slice from series used for volumetry	Free text
Nodule4_Volumetry reliable?	Yes/No	Dropdown
Nodule4_Nodule size (mm3)	Nodule volume	Free text
Nodule4_Nodule maximum diameter (mm)	Nodule longest diameter	Free text
Nodule4_Nodule type	pure ground-glass/part-solid/solid/ IPLN/inflammatory	Dropdown

Field description	Variable input options	Type of input[4]
Nodule4_Lobe	RUL/RML/RLL/LUL/LLL	Dropdown
Nodule4_Position	intraparenchymal/subpleural/endobronchial	Dropdown
Nodule4_Spiculated	No/Yes	Dropdown
Nodule4_ suspicious features	none/ bubble-like appearance/ air bronchogram/ pleural indentation/ pleural retraction/ cyst with irregular wall	Dropdown
(multiple selections possible)		
Nodule4_Brock score[6]	Brock score	Autopopulated
Nodule4_change assessment	Growth (Volume change from baseline >25% if volume reliable=Yes, OR diameter change>2mm if volume reliable=No)/stable/ shrinking/ resolved/NEW	Dropdown
Nodule4_VDT (days)	Volume doubling time from baseline	Free text
**Total number of nodules detected**	0/ 1/ 2/ 3/ 4/ other-free text for maximum number	Dropdown
Emphysema extent[6]	None/mild (<25%)/ moderate (25-50%)/ severe (>50%)	Dropdown
Emphysema predominant type[6]	None/centrilobular/ paraseptal/ panacinar	Dropdown
Highest Brock score	Highest Brock score from four reported nodules	Autopopulated
**Are there incidental pulmonary findings?**	No/ Yes	Dropdown
Bronchiectasis	None/ Mild (airways 1.5- 2X size of artery)/ moderate (airways 2-3X size artery/ severe (>3X size of artery AND >1segment)	Dropdown
Respiratory-Bronchiolitis	Absent/Present	Dropdown
Interstitial lung abnormalities (ILA)	None or ILA other than reticulation/ <5% reticulation of total lung volume/ 5-10% reticulation of total lung volume/ >10% of total lung volume	Dropdown
Infective consolidation	No/ Yes	Dropdown
Active Tuberculosis	No/ Yes	Dropdown
**Are there incidental intrathoracic findings?**	No/ Yes	Dropdown
Mediastinal mass present?	Absent/Present	Dropdown
Mediastinal mass_description	Report position, density and size (use this to describe large lymph nodes that require referral as well)	Free text
Coronary calcification[6]	None/ Mild/ Moderate/ Severe	Dropdown

Field description	Variable input options	Type of input[4]
Aortic valve calcification	None/ Moderate/ Severe	Dropdown
Thoracic Aortic aneurysm	None/ <4cm/ 4.0cm-5.5cm/ >5.5cm	Dropdown
Pleural effusion/thickening or mass	Absent/ Unilateral right/ Unilateral left/bilateral	Dropdown
Pleural effusion or thickening_description	Describe findings (use this to describe unusual lesions eg schwannoma)	Free text
**Are there incidental extrathoracic findings?**	No/Yes	Dropdown
Suspicious Breast lesion	Describe size, position and suspicious feature(s)	Free text
Suspicious thyroid lesion	Describe size, position and suspicious feature(s)	Free text
Liver or splenic lesion	benign/indeterminate and potentially malignant (ill-defined margin, heterogeneous density, mural thickening or nodularity, thick septa)	Dropdown
Liver or splenic lesion_description	Describe size, position and suspicious feature(s)	Free text
Renal lesion	benign (too small to characterise or homogeneous)/ benign (homogeneous -10 to 20HU: thin or imperceptible wall, no mural nodule, septa or calcification)/benign (homogeneous >=70HU : thin or imperceptible wall, no mural nodule, septa or calcification)/benign (solitary, contains ROI <-10HU AND no calcification AND <4cm)/indeterminate and potentially malignant (homogeneous 21-69HU : thin or imperceptible wall, no mural nodule, septa or calcification)/ indeterminate and potentially malignant (heterogeneous, thick or irregular wall, mural nodule, septa or calcification); indeterminate and potentially malignant (solitary, contains ROI <-10HU AND calcification); indeterminate and potentially malignant (multiple, contains ROI <-10HU AND calcification); indeterminate and potentially malignant (solitary AND no calcification AND SIZE >=4cm)	Dropdown
Renal lesion_description	Describe size, position and suspicious feature(s)	Free text
Adrenal lesion	Benign (<10HU and <1cm); indeterminate	Dropdown
Adrenal lesion_description	Describe size, position and suspicious feature(s)	Free text
Abdominal aortic aneurysm	None/ 3-5cm/ >5cm	Dropdown
Bones	None/ osteoporotic fracture <=50%/ osteoporotic fracture >50%/ malignant lytic or sclerotic features	Dropdown

Field description	Variable input options	Type of input[4]
**Is there any other urgent finding?**	No/Yes	Dropdown
Urgent finding description	Description of urgent finding	Free text
Follow up recommendation_nodules	Urgent referral to lung cancer MDT<br>Refer to Screening Review Meeting-specify reason<br>Interval LDCT at 3 months<br>Interval LDCT at 12 months<br>Interval LDCT at 24 months	Dropdown (multiple selections not allowed)<br>Free text for specifying reason
Follow-up recommendation_other	Urgent referral to other cancer MDT- specify which<br>Urgent referral to other non-cancer team-specify which<br>Refer to Chest Clinic<br>Refer to Tuberculosis service<br>GP action required<br>Specify MDT or GP action for incidental finding requiring action, as per NHS England protocol (see Annex 2)	Dropdown (multiple selections allowed)<br>Free text for specifying reason

Annex 2: Protocol for the management of incidental findings in lung cancer screening

Background

Screening for lung cancer with low-radiation dose computed tomography (LDCT) detects thoracic and extrathoracic radiological findings indicative of conditions other than lung cancer. These are termed incidental findings (IFs). IFs may be clinically significant, but it is important to distinguish them from the purpose of the screening programme which is to detect early-stage lung cancer. The reasons for making this distinction are multiple:

- The screening test (LDCT) is not optimised for the detection of IFs and there is no certainty that they will either be sought or found because of this.
- Where there is a threshold for reporting it may be subjectively judged on the LDCT, so reporting may be variable and precision reduced compared with screening-related findings for which the LDCT is optimised.
- There is often insufficient evidence to allow us to know whether IFs detected in asymptomatic participants in a screening programme cause more good than harm[1].
- Investigation of IFs leads to additional costs, which may impair the cost-effectiveness of a screening programme in the absence of associated benefits, although the costs are not necessarily borne by the screening programme.

This document sets out to clarify the above points to maximise potential benefit and minimise harm, according to the available evidence and guidelines[1-4]. The authors are primarily clinicians, and are listed on page 11. This document has been approved by the Lung Cancer Screening Expert Advisory Group.

IFs are a common finding on LDCT with the majority being emphysema (~30% of all findings) and coronary artery calcification (~58% of all findings), both to be expected given the link with smoking[5,6]. IFs may be clinically non-significant and/or not associated with any treatments that lead to beneficial outcomes[5,7]. Identification of IFs may result in investigations that use healthcare resources with limited or no participant benefit. Therefore, it is imperative that processes are in place that minimise referral of and/or action on clinically non-significant IFs, whilst using the opportunity to identify those IFs for which there is a beneficial intervention that improves patient outcomes. Research evidence has not identified a significant lasting impact on quality of life from the detection of IFs[8,9] although indeterminate findings are associated with transient distress[10,11].

National protocol for management of incidental findings

An NHS England Standard Protocol for the Lung Cancer Screening Programme was published in January 2019 and last updated in 2024, it sets out principles for the management of IFs[12] as follows:

- The finding should be clinically significant.
- Clinically non-significant findings should not be reported to the GP or participant.
- There should be agreement between the local screening programme and primary care as to the nature and benefit of the recommended interventions.
- Recommendations for clinical correlation with symptoms by primary care of CT findings should be specific and only made where there is the potential for benefit.

The protocol recommends that IFs should be categorised as:

- Life threatening (warranting direct hospital admission).
- Urgent (mandating urgent referral, including findings indicative of cancer).
- Non-urgent findings (warranting primary or secondary care referral).
- Clinically non-significant findings (that do not require communication and are usually not included in the radiology report, see Table 1)

The European societies for radiology, respiratory medicine, thoracic surgeons and nuclear medicine jointly published a systematic review and clinical practice statement on IFs in lung cancer screening in 2023[1]. This emphasises the importance of establishing which findings have evidence supporting an impact or change to participant management.

Role of Responsible Radiologist

Within each local programme, a Responsible Radiologist (RR) provides ultimate responsibility for the reporting of LDCT scans including the reporting of IFs. The management of the majority of IFs is currently dictated by the LCSP Standard Protocol and Quality Assurance Standards[12,13]. In cases of uncertainty, or where the management of an IF is not specified, radiologists will refer cases to a local multidisciplinary team (MDT) meeting, which should be a dedicated lung cancer screening review meeting (SRM). The RR should lead and/or have oversight of the radiological aspects of such meetings. In this context, and in conjunction with the responsible clinician (RC), the RR will be accountable for decisions made regarding IFs referred to the MDT/SRM.

The RR will also be responsible for quality assurance aspects of radiology reporting, including monitoring of total number and rate of IF reporting on a per radiologist basis and on an aggregate basis within a local programme.

The RR will be responsible for feedback to local reporting radiologists, particularly where individual radiologists are outliers with respect to the number of IFs reported, and/or the reporting of specific IFs. The RR is responsible for the education and training of reporting radiologists, through local governance meetings, screening quality assurance meetings and national external quality assurance. Where individual radiologists are found to be outliers, there should be feedback, potentially re-training and a re-evaluation against guidelines.

Legal aspects

Concern about being subject to litigation may influence the approach to IFs such that clinically non-significant conditions or those without beneficial interventions are more often flagged. This relates to the management of findings that might, potentially, be a source of litigation even though the chance of that is low. This is not the same as a missed lesion (including cancer) where the “miss” is confirmed and confers harm. The latter should be avoided through training and sound radiology practice. The considerations below relate to the decision to flag a detected IF.

LDCT is designed to detect lung cancer and is suboptimal for the detection of many IFs.

In relation to the decision to flag an IF, the two main legal issues that may be open to complaints and negligence claims are firstly the failure to flag an IF that would be harmful if not treated, and treatment would have been possible; and secondly identification of a finding that is itself harmless, but the consequence of identification is harmful.

The consequences of the first issue, in relation to complaints, is that there might be a negligence claim on the grounds that the harm was known, and their failure to disclose/report that information might have delayed the patient's access to treatment.

All staff should be clear about what information the screening test can and cannot find, and the reliability of that finding. The sensitivity of the screening tool will be reviewed periodically such that new potential findings can be considered and a process for reporting can be devised if appropriate. By following this protocol, the chance of a successful negligence claim will be reduced.

The second issue gives rise to complaints that physical and / or psychological harm arose from the disclosure of an IF. Even were this to be the case, and the disclosure of the IF was in breach of a duty of care, the harm that arose – whether physical or psychological – would have to be causally linked to the disclosure of the IF.

IFs identified through asymptomatic screening should be communicated to patients using methods appropriate to the potential significance to health. For common findings this can be by standardised letter. For more serious or urgent findings this should be by direct communication by secondary care to avoid delay, with communication with primary care. For clinically non-significant findings, or those that do not require additional action, no communication is usually appropriate.

Table 1 shows a list of IFs potentially identified through screening, how reliable those findings are, reporting and management recommendations and the consequences for a participant. This is based on the principles outlined in Section 2 and is designed to reflect the best balance of benefit vs. harms that, coupled with IF specific information to patients, will mitigate complaints and make any negligence claim unlikely.

There must be a very clear consent process, where the details are made understandable to participants. Key points are:

- It should be clear exactly what the screening process can find, and what it cannot;
- It should state what results will be communicated to participants and what results are less likely to be communicated;
- It should indicate the level of reliability that can be attributed to the results;
- It must be written in sufficiently lay language, that there is a reasonable expectation that the concept of risk and what the screening process involves (including the possibility of receiving IFs) is understood by the participants so that informed decision making can be supported[14].

If there are incidental findings where the result can be relied on, then the obligation to act on the findings is determined according to whether:

- the finding is harmful;
- there is a recommended change in clinical management that would follow from this finding.

Any finding that results in a Duty of Candour issue should be approached via a defined process, as in other screening programmes. This may include IFs. Guidance on duty of candour in NHS screening programme can be found here: https://www.gov.uk/government/publications/nhs-screening-programmes-duty-of-candour

Any IF reported by a radiologist may be added to the patient's record, regardless of whether the radiologist or MDT/SRM determine it to be clinically significant or clinically non-significant, and whether the IF is communicated to the patient. All individuals have the legal right to access information held about them by health and care organisations, usually through a Subject Access Request. Thus, participants may become aware of clinically non-significant diagnoses that were not previously disclosed to them. It is important that participants receive information about this possibility and the reasons that they may not be informed as part of the consent process.

It is recommended that this protocol is referenced in any clinically non-significant additions to the patient record by the lung cancer screening programme that are not communicated to patients.

Role of NHSE/national team

NHS England (NHSE) is responsible for performance metrics for the programme. This includes the management information that must be submitted by local and regional systems so the NHSE national team can monitor programme performance. Included in these performance metrics are incidental findings referrals. In addition, the programme clinical dataset suggests a larger number of data metrics that individual areas are recommended to collect but does not need to be submitted to the national team. These metrics are periodically updated. Currently, the NHSE Quality Assurance Standards stipulate overall rates of clinically significant incidental findings should be <8%.

Approach to outliers

Once outliers are identified, feedback can be provided to responsible and individual radiologists and SRMs for education and calibration.

Table 1: Incidental findings, reporting and management, based on latest evidence*.

This list is not exhaustive and other, less common findings, may be reported according to normal radiological practice.
The evidence for this table was based on a recent systematic review of IF in LDCT screening[1],

FINDING	Reliability of detection and characterisation by LDCT (Low Dose Computed Tomography)	Radiology report and management recommendations<br>If reporting is recommended this means that the chance of the finding being harmful is significant, although any action may be associated with both benefit and harm		Potential consequences for participant (benefit / ***harm***)
		**Report content**	**Management if baseline or new**	
**PULMONARY**				
**Emphysema**	Good	Classify as:<br>mild (<25%)<br>moderate (25-50%)<br>severe >50%.	Inform participants with moderate to severe radiological emphysema about the findings and recommend they seek advice from primary care if they have symptoms.<br><br>Do not refer participants with known diagnosis of COPD or if LHC establishes the participant is not impacted by dyspnoea and or cough.	Early diagnosis and treatment of symptomatic COPD.<br>Increased incentive to quit smoking.<br>*No benefit and extra worry where no symptoms or no response to treatment.*

			Smoking cessation referral for all current smokers. Further emphasis on smoking cessation in results letter in those with moderate or severe emphysema.	
**Interstitial lung abnormalities (ILA)**	Good	Report all ILA as an estimated percentage of whole lungs	Further scanning within the lung cancer screening programme may flag progression for all ILA not referred.<br>ILA involving more than 10% of either the whole lungs should be referred for specialist review / reviewed at the Screening Review Meeting (SRM).	Early diagnosis and treatment of ILD.<br>*No benefit and extra worry*. *Unnecessary treatments and investigations* with no benefit.
**Bronchiectasis**	Good	Report bronchiectasis when moderate or severe (more than 2X the diameter of the artery and involving more than one segment).	Review at screening review meeting if moderate or severe.<br><br>Ensure clinical assessment to check for symptoms either via existing records, lung health check or direct assessment in primary or secondary care.	Identification and treatment of symptoms including prompting early treatment of future infections.<br>Identification of an underlying cause.<br>*No benefit if asymptomatic and not underlying cause. Unnecessary treatment.*

**Respiratory bronchiolitis (RB)**	Good	Do not report.	Smoking cessation will be offered to all current smokers irrespective of the presence of RB-ILD.	
**Consolidation**	Good	Classify as:<br>Possibly inflammatory possibly malignant	If cancer more likely than inflammation refer to SRM.<br><br>Inflammation more likely than cancer refer to SRM consider repeat CT Repeat CT at 6 weeks or 3 months depending on concern (within or outside screening programme).<br><br>Do not report minor areas of consolidation or tree in bud that are clearly inflammatory.	Early identification and treatment of malignancy / other diagnosis.<br>*Unnecessary worry, further imaging or work up and treatment for self-limiting resolving lesions.*
**Pleural effusion/ thickening**	Moderate	Report size and laterality and whether malignant features present.	Refer directly via SRM for clinical assessment and work-up if suspicious appearances including a new effusion, pleural thickening suspicious for malignancy or mass lesion.<br>This includes schwannomas.	Early identification and treatment of malignancy / other diagnosis.<br>*Unnecessary worry, further imaging or work up for benign findings.*

**Pleural plaques**	Good	Do not report, or do so only as a note. Reporting is only recommended in context of screening where compensation is available (i.e. Scotland and Northern Ireland	No clinical activity should be generated for benign appearances.	In some UK countries, compensation is available to people exposed to asbestos who have pleural plaques.
**Tuberculosis (TB)**	Good	Report if active TB likely and differential diagnoses.	Referral into local TB service.	Opportunity for treatment and contact tracing.
**Bronchial wall thickening**	Good	Do not report.	No action required.	
**Coronary calcification (CAC)**<br>Note: all participants should have had a Q-risk or similar assessment.	Good	Report CAC, classify (using simple visual scoring) as:<br>Mild<br>Moderate<br>Severe	Cardiovascular (CV) risk assessment reminder if moderate or severe CAC present, unless already on lipid lowering therapy or known to have ischaemic heart disease.<br>Note: it is not established that mild CAC confers extra risk over Q-risk or similar assessment.	Should provide an extra prompt for CV risk assessment[15] in those at markedly increased risk of CV events. However with current entry criteria, almost all participants will be eligible for primary prevention regardless of coronary calcification.
**Aortic valve disease**	Good	Report aortic valve calcification (AVC) if moderate or severe.<br>Classify using simple visual scoring.<br>Isolated specks of calcification do not require reporting.	Refer those with moderate or severe AVC for evaluation with echocardiography via SRM.	Earlier assessment of aortic valve disease<br>*No benefit and extra worry, unnecessary further investigations.*

**Thoracic aortic calcification/ dilatation**	Good	Do not report thoracic aortic calcification. Report thoracic aorta diameter if ≥45mm.	Referral for further assessment for those with thoracic aorta > 45mm diameter according to local guidelines/ pathways; if >50mm urgent referral.	Earlier option for medical treatment / monitoring / surgical intervention *No benefit in outcome, extra worry / harm from work-up*
**Mediastinal mass**	Moderate	Report size, morphology, position, and density / texture, including whether cystic.	Refer all non-cystic lesions to SRM.<br><br>Options for management include surveillance as part of the screening programme **or** work-up depending on clinical assessment.	Early identification of malignant or harmful lesion. *Extra worry and work up for benign disease.*
**Mediastinal lymph nodes**	Moderate	Report mediastinal and hilar lymphadenopathy ≥ 15mm short axis.	Refer to SRM for further assessment.	Early diagnosis of significant disease. *Worry and work up for harmless findings.*
**Thyroid abnormalities**	Poor	Report nodules with suspicious features such as local lymphadenopathy, punctate microcalcification.	Refer to thyroid MDT via SRM for nodules with suspicious features that are ≥ 20mm.	Early diagnosis of thyroid cancer. *Work up of benign or indolent disease.*
**Cardiac decompensation / pericardial effusion**	Moderate	Report moderate or large pericardial effusion. Report features of significant decompensation.	Referral for echocardiography and clinical assessment via SRM or primary care; urgent referral may be indicated for concerning features.	Early diagnosis and treatment of pericardial disease / cardiac failure *Unnecessary activity if already known*

**Oesophageal lesions**	Moderate	Report diffuse wall thickening, or focal lesions.	Referral for further assessment via SRM.	Early diagnosis of oesophageal disease.<br>*Unnecessary work up and worry for no significant disease or normal findings.*
**Abdominal aortic aneurysm (AAA)**	Moderate	Report all AAA.	Referral for further assessment / surveillance according to guidelines;<br>3-5cm, referral<br>>5cm, urgent referral.	Early identification of AAA.
**Breast nodules**	Moderate	Report size, site, calcification, density, and interval change.	Refer any breast lesion (via SRM) that is NOT clearly benign, (i.e., stable, well-defined margins or multiple) to the breast service unless already known.	Early diagnosis of breast cancer<br>*Overdiagnosis; worry and harm from benign disease.*
**Liver lesions**	Poor<br>(Including partial imaging)	Report size and attenuation<br>Benign features: sharp margin and homogenous low attenuation (≤ 20 Hounsfield Unit (HU)), (focal) fatty sparing or deposition do not require further investigation or reporting<br>Incompletely imaged lesions or lesions too small to characterize should not by itself	Lesions < 1cm: no further investigation<br>Lesions ≥ 1cm and no benign features: referral via SRM:<br>refer malignant lesions to the appropriate cancer pathway<br>indeterminate lesions consider further investigation with CE CT/ ultrasound/ MRI.	Diagnosis of primary or secondary cancer.<br>*Unnecessary worry, and work up for non-significant disease.*

		prompt further investigation.		
**Renal lesions**	Poor (Including partial imaging)	Report size, site, attenuation, calcification Classify as malignant, indeterminate, and benign or incompletely imaged/ unable to evaluate. Incompletely imaged lesions or lesions too small to characterize should not by itself prompt further investigation.	Homogenous hypodense cysts do not require further investigation. Soft tissue, hyperdense or mixed density renal mass >1cm – or masses >3cm that show growth in comparison with prior imaging if available refer to SRM.	Diagnosis of primary or secondary cancer. *Unnecessary worry, and work up for non-significant disease.*
**Bone abnormalities**	Moderate	Report >50% loss of vertebral height in at least one vertebra.<br><br>Report any lesions suspicious for malignancy.	Refer via SRM to primary care or osteoporosis service for >50% loss of vertebral height<br><br>Refer to SRM.	Prevention of fracture. *Worry and inconvenience.*
**Adrenal lesions**	Moderate	Report size and attenuation Lesions < 10mm or <10HU in density and 10-40mm diameter do not require reporting.	Refer to SRM lesions which are >10-40mm diameter with attenuation >10HU, or lesions with these characteristics growing on serial scans. Refer lesions >40mm	Early identification of adrenal disease *Worry and work up of non-significant lesion.*

This table assumes no clinical information is available. *Review recommended for new evidence January 2026

Authors:

LCSP Expert Advisory Group
David R Baldwin
Arjun Nair
Richard Lee
Matthew Callister
Ruth Stirton
Tim Windle
Anne Mackie
Liz Rochelle
Alan Bagnall
Anand Devaraj
Emily Bartlett
Emma O'Dowd

Protocol for the management of incidental findings in lung cancer screening - references

1. O'Dowd EL, Tietzova I, Bartlett E, et al. ERS/ESTS/ESTRO/ESR/ESTI/EFOMP statement on management of incidental findings from low dose CT screening for lung cancer. Eur Respir J 2023. DOI: 10.1183/13993003.00533-2023.
2. Williams MC, Abbas A, Tirr E, et al. Reporting incidental coronary, aortic valve and cardiac calcification on non-gated thoracic computed tomography, a consensus statement from the BSCI/BSCCT and BSTI. Br J Radiol 2021;94(1117):20200894. DOI: 10.1259/bjr.20200894.
3. Munden RF, Carter BW, Chiles C, et al. Managing Incidental Findings on Thoracic CT: Mediastinal and Cardiovascular Findings. A White Paper of the ACR Incidental Findings Committee. J Am Coll Radiol 2018;15(8):1087-1096. DOI: 10.1016/j.jacr.2018.04.029.
4. Radiology ACo. ACR Lung Cancer Screening CT Incidental Findings Quick reference Guide. (https://www.acr.org/-/media/ACR/Files/Lung-Cancer-Screening-Resources/LCS-Incidental-Findings-Quick-Guide.pdf).
5. Bartlett EC, Belsey J, Derbyshire J, et al. Implications of incidental findings from lung screening for primary care: data from a UK pilot. NPJ Prim Care Respir Med 2021;31(1):36. DOI: 10.1038/s41533-021-00246-8.
6. Bradley P, Bola BM, Balata H, Sharman A, Booton R, Crosbie PAJ. Incidental findings in low dose CT lung cancer screening of high-risk smokers: Results from the Manchester lung Health Check pilot. Lung Cancer 2022;173:1-4. DOI: 10.1016/j.lungcan.2022.08.017.
7. Gareen IF, Gutman R, Sicks J, et al. Significant Incidental Findings in the National Lung Screening Trial. JAMA Intern Med 2023;183(7):677-684. DOI: 10.1001/jamainternmed.2023.1116.
8. Clark SD, Reuland DS, Brenner AT, Jonas DE. Effect of Incidental Findings Information on Lung Cancer Screening Intent: a Randomized Controlled Trial. J Gen Intern Med 2022;37(14):3676-3683. DOI: 10.1007/s11606-022-07409-4.
9. Gareen IF, Duan F, Greco EM, et al. Impact of lung cancer screening results on participant health-related quality of life and state anxiety in the National Lung Screening Trial. Cancer 2014;120(21):3401-9. DOI: 10.1002/cncr.28833.
10. Brain K, Lifford KJ, Carter B, et al. Long-term psychosocial outcomes of low-dose CT screening: results of the UK Lung Cancer Screening randomised controlled trial. Thorax 2016;71(11):996-1005. DOI: 10.1136/thoraxjnl-2016-208283.
11. Kummer S, Waller J, Ruparel M, Duffy SW, Janes SM, Quaife SL. Psychological outcomes of low-dose CT lung cancer screening in a multisite demonstration screening pilot: the Lung Screen Uptake Trial (LSUT). Thorax 2020;75(12):1065-1073. DOI: 10.1136/thoraxjnl-2020-215054.
12. National Health Service England, - National, Cancer, Programme. Targeted Screening for Lung Cancer with Low Radiation Dose Computed Tomography. Standard Protocol prepared for the Lung Cancer Screening Programme. (https://www.england.nhs.uk/publication/targeted-screening-for-lung-cancer/).

13. National Health Service. England. Targeted Screening for Lung Cancer with Low Radiation Dose Computed Tomography. Quality Assurance Standards prepared for the Lung Cancer Screening Programme. (https://www.england.nhs.uk/wp-content/uploads/2019/02/B1647-quality-assurance-standards-targeted-lung-health-checks-programme-v2.pdf).
14. Hotson v East Berkshire AHA. House of Lords; 1987.
15. Mamudu HM, Paul TK, Veeranki SP, Budoff M. The effects of coronary artery calcium screening on behavioral modification, risk perception, and medication adherence among asymptomatic adults: a systematic review. Atherosclerosis 2014;236(2):338-50. DOI: 10.1016/j.atherosclerosis.2014.07.022.

Annex 3: National incidental findings pathways for targeted lung cancer screening

Locally agreed pathways for the management of incidental findings are important to maximise efficiency whilst bringing any benefits to participants that may result from detection. All incidental findings flagged by LDCT readers should be discussed at the Screening Review Meeting (SRM) where previous imaging and clinical factors can be included in the assessment. If the SRM is unsure of the significance of a finding outside it specialism then advice should be sought from the relevant speciality prior to any action. The SRM should request tests that are required to determine if a referral needs to be made, according to local agreements (e.g. echocardiogram for aortic valve calcification).

All participants should have received at the time of consent, and have ongoing access to, detailed information about incidental findings. This is important for the participant to understand why the findings have or have not been communicated to them and why action is or is not recommended.

Most findings and actions can be communicated by letters in a standardised format to the participant with copies to primary care. Bespoke letters may be needed to communicate outcomes of additional investigations or where a standard letter is not available. Standard letters and return to screen outcomes can be managed by the screening admin team.

The requesting of investigations and certain referrals may need to be done by medical staff deputising for the Responsible Clinician. In general, sites should avoid asking primary care to action incidental findings although this can vary according to local agreements. Alternative pathways are given in the table where primary care may be involved; “No alternative pathway” indicates the finding should be managed by secondary care or within the programme. Where alternative pathways include extra investigations and interventions, there should be in-service evaluation.

Where participants will exit screening (e.g. for age reasons), consideration can be given to act upon more significant incidental findings which otherwise would have been reasonable to survey at incident rounds. When participants are known to have new or existing comorbidities that mean they may not benefit in continuing screening, this should be discussed with the participant and a shared decision about continuation in the programme reached.

Programmes are encouraged to work closely with primary and secondary care teams to avoid uncertainty in onward referral.

FINDING	Pathway Recommendations<br>Review at Screening Review Meeting or Triage<br>(Referral may not be required if finding is already known; all participants returned to screening via the screening coordinator)		Communications and responsibilities<br>SC = Screening Coordinator<br>RC = Responsible Clinician or deputy	
	**Preferred**	**Alternative**	**Format and Content**<br>**SL** = standard letter;<br>**BL** = bespoke letter.	**Responsibl e**
**Emphysema (action required for moderate or severe only)**	Communication only.	Locally agreed and funded services may offer assessment in either primary or secondary care. This may include assessment of symptoms and integration of spirometry.	**SL**<br>Inform participants about the findings.<br>Recommend they seek advice from primary care if they have symptoms.<br>Encourage smoking cessation.	**SC**
**Interstitial lung abnormalities (ILA)**	1. Referral by the screening service direct to the local ILD service for ILA >10% or for that which has progressed.<br>2. For <10% return to screen or discharge, with standard letter.<br>If concern and exiting the programme, referral to ILD service for potential ongoing surveillance	Referral to primary care for onward referral to ILD service. (Same thresholds as for preferred pathway.)	**SL**<br>Explain the referral and the finding.<br>Discharge letter if no further screening	**SC**<br>**RC**

**Bronchiectasis**	Direct referral to local respiratory / bronchiectasis service for moderate or severe disease.	Referral to primary care for clinical assessment and onward referral.	**SL**<br>to participants communicating the referral and the finding.	**SC**<br>**RC**
**Consolidation**	SRM discussion regarding likelihood of malignant vs. benign aetiology then<br>1. Urgent cancer pathway referral for findings suspicious of cancer.<br>2. Interval CT if findings more suggestive of infection, either within programme or in secondary care.<br>3. Primary care clinical review only if a decision about antibiotics is needed.	SRM discussion regarding likelihood of malignant vs. benign aetiology then<br><br>Referral to primary care for clinical assessment and onward referral if malignancy unlikely.	**SL**<br>to participants communicating the referral and the finding.<br>**BL**<br>follow-up letter with outcome	**SC**<br><br><br>**RC**
**(Pleural plaques)**	(In Scotland and Northern Ireland participant needs to be informed. A flag for SRM discussion is only needed in these countries.)		**(SL**<br>Emphasise that plaques are benign.<br>Compensation may be available as they result from asbestos exposure.)	**(SC)**
**Tuberculosis (TB)**	Direct referral to local TB service for active TB	Referral into local TB service via primary care	**SL**<br>to participant	**SC**<br>**RC**
**Coronary artery calcification (CAC)**	No referral.<br>Letter to participant with moderate or severe CAC to encourage primary prevention and lifestyle change including seeking medical advice if symptoms.	Where local service funded there may be a trigger for primary care review and active intervention.	**SL**<br>to participant about the finding, eligibility for lipid lowering therapy, and need to seek medical advice for symptoms.	**SC**

			Include smoking cessation and other lifestyle advice.	
**Aortic valve calcification (with no previous echocardiogram)**	Echocardiogram requested in secondary care for moderate and severe calcification and only referred to cardiology if significant aortic valve disease – may simply be added to the local valve register.	Refer to primary care for moderate and severe calcification to request echocardiogram and onward management.	**SL**<br>Reason for echocardiogram<br>**BL** or **SL**<br>Outcome.	**SC**<br><br>**RC**
**Thoracic aortic dilatation**	Aorta >45-50mm return to screen and check BP in primary care<br>>50mm urgent referral to cardiology	No alternative pathway	**SL**<br>1. Aorta >45-50mm: About finding and need to check blood pressure.<br>2. For >50mm, standard letter about finding and referral	**SC**<br>**RC**
**Mediastinal mass (non-cystic)**	Options for management include surveillance as part of the screening programme **or** work-up depending on clinical assessment.	No alternative pathway	**SL**<br>Explain finding.<br>Explain plan.<br>**BL** for outcome of further imaging	**SC**<br><br>**RC**
**Suspected Cancer (includes Thyroid, oesophageal, pleural, renal,**	1. Clear evidence of cancer on LDCT: direct referral to cancer service either via site specific MDT or cancer upgrade.<br>2. Possible evidence of cancer on LDCT but further investigation needed to	No alternative pathway	**SL**<br>Inform about urgent referral<br><br>**BL**	**SC**<br><br>**RC**

**liver, breast, adrenal, bone)**	clarify whether referral is needed: direct referral for further imaging and review.<br>3. See incidental findings protocol for thresholds		Inform and explain further imaging and outcome	
**Mediastinal lymph nodes**	If referral required (for nodes ^{3}15mm short axis), manage within respiratory service.	No alternative pathway	**SL**<br>Inform about referral.	**SC**
**Cardiac decompensation / pericardial effusion**	Discussion at SRM<br>1. Significant pericardial effusion – manage in secondary care (echocardiogram).<br>2. For cardiac decompensation clinical assessment in primary care.	Manage both scenarios in secondary care	**SL**<br>Inform about echocardiogram<br>**BL**<br>Findings, and outcome	**SC**<br><br>**RC**
**Abdominal aortic aneurysm (AAA)**	Refer to vascular team as follows:<br>3-5cm, referral<br>>5cm, urgent referral.	Referral for primary care to action with clear recommendation (unless >5cm).	**SL**<br>Findings and need for referral	**SC**<br><br>**RC**
**Osteoporosis (fracture > 50% of vertebral height)**	Refer direct to osteoporosis service.	Refer to primary care for management.	**SL**	**SC**<br><br>**RC**

QA Standards Authors

Prof David Baldwin	Consultant Respiratory Physician, Nottingham University Hospital
Dr Claire Bloomfield	Chief Operating Officer for Medical Imaging, University of Oxford
Dr Matthew Callister	Consultant Respiratory Physician, Leeds Teaching Hospitals NHS Trust
Dr Yan Chen	Associate Professor of Cancer Screening, School of Medicine at University of Nottingham
Dr Philip Crosbie	Senior Lecturer of Respiratory, Manchester University
Prof Anand Devaraj	Consultant Thoracic Radiologist, Royal Brompton Hospital
Dr Jesme Fox	Medical Director, Roy Castle Lung Cancer Foundation
Prof Fergus Gleeson	Professor of Radiology, Oxford University
Dr Samanjit Hare	Consultant Radiologist, Barnet General Hospital
Nicola Keat	Head of Clinical Research Groups, National Cancer Research Institute
Prof Sam Janes	Consultant Respiratory Physician, University College London Hospitals NHS Foundation Trust
Dr Jodie Moffat	Head of Early Diagnosis, Cancer Research UK
Dr Arjun Nair	Consultant Thoracic Radiologist, University College London Hospitals NHS Foundation Trust
Dr Nicholas Screaton	Consultant Radiologist, Royal Papworth NHS Foundation Trust
Dr Anna Sharman	Consultant Thoracic Radiologist, Manchester University NHS Foundation Trust
Dr Nicola Strickland	Consultant Radiologist, Imperial College Healthcare NHS Trust
Dr Alexis Webb	Cancer Research UK
Dr Richard Lee	Consultant Respiratory Physician, The Royal Marsden NHS Foundation Trust

Reviewed by CT Screening Advisory Sub-Group of the Lung Cancer Clinical Expert Group.

References

1. Berlin, L., *The incidentaloma: a medicolegal dilemma.* Radiol Clin North Am, 2011. **49**(2): p. 245-55.

2. van de Wiel, J.C., et al., *Neglectable benefit of searching for incidental findings in the Dutch-Belgian lung cancer screening trial (NELSON) using low dose multidetector CT.* Eur Radiol, 2007. **17**(6): p. 1474-82.

3. Booth, T.C., *Incidental findings on imaging.* BMJ, 2018. **361**: p. k2611.

4. RCR, *Management of Incidental Findings Detected During Research Imaging.* RCR publications, 2011.

5. Ding, A., J.D. Eisenberg, and P.V. Pandharipande, *The economic burden of incidentally detected findings.* Radiol Clin North Am, 2011. **49**(2): p. 257-65.

6. Berland, L.L., et al., *Managing incidental findings on abdominal CT: white paper of the ACR incidental findings committee.* J Am Coll Radiol, 2010. **7**(10): p. 754-73.

7. Roberts, H.R., et al., *Airflow obstruction in bronchiectasis: correlation between computed tomography features and pulmonary function tests.* Thorax, 2000. **55**(3): p. 198-204.

8. Doyle, T.J., et al., *Interstitial lung abnormalities and reduced exercise capacity.* Am J Respir Crit Care Med, 2012. **185**(7): p. 756-62.

9. Munden, R.F., et al., *Managing Incidental Findings on Thoracic CT: Mediastinal and Cardiovascular Findings. A White Paper of the ACR Incidental Findings Committee.* J Am Coll Radiol, 2018. **15**(8): p. 1087-1096.

10. Chiles, C., et al., *Association of Coronary Artery Calcification and Mortality in the National Lung Screening Trial: A Comparison of Three Scoring Methods.* Radiology, 2015. **276**(1): p. 82-90.

11. Raju, P., et al., *Aortic valve calcification - a commonly observed but frequently ignored finding during CT scanning of the chest.* Int J Clin Pract, 2012. **66**(6): p. 552-5.

12. Gore, R.M., et al., *Management of Incidental Liver Lesions on CT: A White Paper of the ACR Incidental Findings Committee.* J Am Coll Radiol, 2017. **14**(11): p. 1429-1437.

13. Herts, B.R., et al., *Management of the Incidental Renal Mass on CT: A White Paper of the ACR Incidental Findings Committee.* J Am Coll Radiol, 2018. **15**(2): p. 264-273.

14. Lee, J. H., S.Y. Jeong, and Y.H. Kim, *Clinical significance of incidental thyroid nodules identified on low-dose CT for lung cancer screening.*

15. Multidiscip Respir Med, 2013. **8**(1): p. 56.

16. Field JK, Duffy SW, Baldwin DR, Brain KE, Devaraj A, Eisen T, Green BA, Holemans JA, Kavanagh T, Kerr KM, et al: *The UK Lung Cancer Screening Trial: a pilot randomised controlled trial of low-dose computed tomography screening for the early detection of lung cancer*. Health Technol Assess 2016, 20:177.

17. Liverpool Healthy Lung Programme – Second year Evaluation Report. Available at: https://www.liverpoolccg.nhs.uk/media/3245/final-lhlp-2nd-year-report-10-july-2018-with- logos.pdf

18. Crosbie PA, Balata H, Evison M, Atack M, Bayliss-Brideaux V, Colligan D, Duerden R, Eaglesfield J, Edwards T, Elton P, et al: *Implementing lung cancer screening: baseline results from a community-based ‘Lung Health Check’ pilot in deprived areas of Manchester*. Thorax 2018.

19. Van de Wiel JC, Wang Y, Xu DM, Van der Zaag-Loonen HJ, Van der Jagt EJ, Van Klaveren RJ, Oudkerk M, group Ns: *Neglectable benefit of searching for incidental findings in the Dutch-Belgian lung cancer screening trial using low-dose multidetector CT*. Eur Radiol 2007, 17:1474-1482.

NHS England
Wellington House
133-155 Waterloo Road
London
SE1 8UG

Contact: enquiries@england.nhs.uk

This publication can be made available in a number of alternative formats on request.

© NHS England 2022 | PR1647
